# *Exo*-Functionalized Metallacages as Host-Guest Systems for the Anticancer Drug Cisplatin

**DOI:** 10.3389/fchem.2019.00068

**Published:** 2019-02-18

**Authors:** Ben Woods, Margot N. Wenzel, Thomas Williams, Sophie R. Thomas, Robert L. Jenkins, Angela Casini

**Affiliations:** School of Chemistry, Cardiff University, Cardiff, United Kingdom

**Keywords:** metallacages, supramolecular coordination complexes, water solubility, host-guest chemistry, cisplatin

## Abstract

Within the framework of designing new self-assembled metallosupramolecular architectures for drug delivery, seven [Pd_2_L_4_]^4+^ metallacages (L = 2,6-bis(pyridine-3-ylethynyl)pyridine) featuring different groups in *exo*-position, selected to enhance the cage solubility in aqueous environment, were synthesized. Thus, carboxylic acids, sugars, and PEG groups were tethered to the bispyridyl ligands directly or via disulfide bond formation, as well as via click chemistry. The ligands and respective cages were characterized by different methods, including NMR spectroscopy and high-resolution electrospray mass spectrometry (HR-ESI-MS). While the two ligands featuring carboxylic acid-functionalized groups showed improved solubility in water, the other ligands were soluble only in organic solvents. Unfortunately, all the respective self-assembled cages were also insoluble in water. Afterwards, the encapsulation properties of the anticancer drug cisplatin in selected [Pd_2_L_4_]X_4_ cages (X = ${\text{NO}}_{3}^{-}$, ${\text{BF}}_{4}^{-}$) were studied by ^1^H, ^1^H DOSY, and ^195^Pt NMR spectroscopy. The effect of the counter ions as well as of the polarity of the solvent in the drug encapsulation process were also investigated, and provided useful information on the host-guest properties of these experimental drug delivery systems. Our results provide further experimental support for previous studies that suggest the desolvation of guests from surrounding solvent molecules and the resulting solvent rearrangement may actually be the primary driving force for determining guest binding affinities in metallacages, in the absence of specific functional group interactions.

## Introduction

Supramolecular chemistry has its roots in biology, and in recent years the unique and often advantageous properties of supramolecular materials have led to their extensive exploration in the fields of biomolecular recognition, drug delivery, disease diagnosis, and imaging. In this context, supramolecular coordination complexes (SCCs) hold great promise (Cook et al., 2013; Casini et al., 2017). In fact, the number of reports on the bioactivity of three-dimensional (3D) SCCs with different shapes has substantially increased (Kaner et al., 2016; Preston et al., 2016; Casini et al., 2017) and includes helicates (Schmitt et al., 2012), metallacages (Vajpayee et al., 2011), cubes (Ahmedova et al., 2016), prisms(Ahmad et al., 2015), and capsules (Therrien, 2013). These systems are of general formula M_n_L_m_, where M is usually Fe(II), Pd(II), Pt(II), or half-sandwich organometallic clips based on Ru(II), Os(II), or Ir(III) and Rh(III), and L is the ligand of the coordination complex.

In 2012, Lewis et al. reported on the encapsulation properties of the anticancer drug cisplatin within cationic [Pd_2_L_4_]^4+^ cages (L = 2,6-bis(pyridine-3-ylethynyl)pyridine as the bidentate ligand) studied by NMR and X-ray diffraction analysis (Lewis et al., 2012). Following these promising results, as part of our efforts to develop functional metallosupramolecular drug delivery vectors, we have recently expanded the family of *exo*-functionalized [Pd_2_L_4_]^4+^ cages coupled to fluorescent groups for imaging by fluorescence microscopy (Kaiser et al., 2016; Schmidt et al., 2016a,b), as well as to peptides for targeted drug delivery of cisplatin to cancer cells (Han et al., 2017, 2018). It is worth mentioning that while cisplatin occupies a crucial role in the treatment of various malignant tumors, its efficacy, and applicability are heavily restricted by severe systemic toxicities and drug resistance. Thus, our recent studies have exploited the host-guest properties of targeted metallacages to enhance the activity of cisplatin in cancer cells, protecting it from metabolism (Han et al., 2017). Of note, the reduced toxicity of cisplatin encapsulated in integrin targeted metallacages has also been demonstrated by us in an *ex vivo* model (Han et al., 2017).

**Scheme 1:**
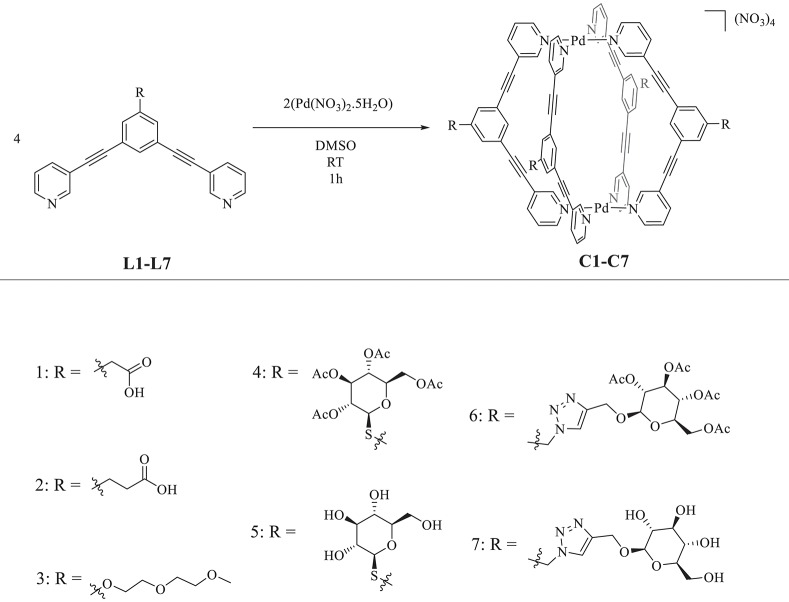
Top: General scheme for [Pd_2_L_4_]^4+^ metallacage formation via self-assembly. Bottom: Numbered (n) chemical structures of the *exo*-functionalized groups envisioned to improve the cages' aqueous solubility.

As the initially developed cage architectures displayed scarce water solubility, we were interested in improving their hydrophilicity via derivatization of the ligand scaffold. Thus, here, a small library of bis(pyridyl) ligands—of general scaffold 3,5-bis(3-ethynylpyridine)phenyl (Scheme 1) - *exo*-functionalized with chemical groups envisioned to improve the aqueous solubility, were synthesized and self-assembled into the corresponding [Pd_2_L_4_]^4+^ cages. The products were characterized by NMR, HR-ESI-MS, and IR. The solubility of the resulting metallacages was then tested in water, as well as in a range of common organic solvents. Furthermore, in order to elucidate the host-guest chemistry of the metallacages and the factors that favor cisplatin's retention in the supramolecular complexes, essential aspects to exploit the cages as drug delivery systems, the drug encapsulation was studied by ^1^H, ^1^H DOSY, and ^195^Pt NMR spectroscopy under different conditions.

## Results and Discussion

Adapting previously reported procedures (Schmidt et al., 2016b), the synthesis of the *exo*-functionalized ligand scaffold with carboxylic acid groups, **L1**, and **L2** was first achieved by Sonogashira cross-coupling of a benzyl-protected 1,3-dibromophenyl precursor with 3-ethynylpyridine, followed by basic deprotection of the benzyl ester to afford the free acid, forming the highly conjugated bispyridyl ligand scaffold in good yields (Scheme S1). A polyethylene glycol *exo*-functionalized ligand (**L3**) was also synthesized. In this case, a ligand bearing a central phenol aromatic ring was synthesized via Sonogashira cross-coupling, which was then was coupled to 1-chloro-2-(2-methoxyethoxy) ethane under basic conditions to achieve ligand **L3** (Scheme S2). In addition, ligands **L4** and **L5**, featuring water-soluble thio-β-D-glucose moieties, were synthesized for the first time. This was achieved by first synthesizing an alkyl bromide *exo*-functionalized ligand via classic Appel reaction using triphenylphosphine, carbon tetrabromide, and 3,5-bis(3-ethynylpyridine)-benzylalcohol; the latter having been synthesized via Sonogashira cross-coupling (Scheme S3). The 1-thio-β-D-glucose tetraacetate group was then conjugated under basic conditions following a S_N_2 type reaction to afford the ligand **L4**. Ligand **L5** was achieved by deacetylation of **L4** using Amberlite-IRA 401 (OH^−^) ion exchange beads, which were stirred together at room temperature overnight, following previously reported procedures (Lewis et al., 2014). Finally, the ligands **L6** and **L7** were synthesized using standard CuAAC “Click” reaction conditions (Scheme S4) following similar procedures reported for analogous tris-pyridyl ligand scaffolds (Lewis et al., 2013). Noteworthy, the latter were reported to be soluble in water, and therefore, offered a logical starting point for functionalization of the herein selected 3,5-bis(3-ethynylpyridine) phenyl ligands. All the ligands were characterized by NMR (^1^H, ^13^C) and HR-ESI-MS. A summary of the synthesized ligands and cages can be found in Scheme S5.

The solubility of the ligands in water was assessed by overnight sonication in D_2_O (10 mg/mL, 20°C), before filtration (pore size 450 μm). The filtrate was analyzed by ^1^H NMR to determine if detectable amounts of ligand were present. Unfortunately, the *exo*-functionalized bispyridyl ligand scaffolds were found to be scarcely soluble, and were all undetectable in D_2_O solution, except for the carboxylic acid functionalized ligands (**L1** = 10 mg/mL, **L2** > 100 mg/mL). The latter could be analyzed by ^1^H NMR in D_2_O (data not shown).

Afterwards, [Pd_2_L_4_]^4+^ cage formation was achieved via self-assembly by reacting two equivalents of Pd(II) nitrate pentahydrate with four equivalents of the functionalized ligand (**L1-L7**) in DMSO at room temperature, to form the corresponding homoleptic metallacages (**C1-C7**) (Scheme 1). The products were analyzed by NMR (^1^H, ^13^C, ^11^B, and ^19^F), HR-ESI-MS (Figures S1–S3, S5–S9) and IR. Following previously reported examples (Schmidt et al., 2016c), quantitative cage formation was monitored by ^1^H NMR, whereby the peaks corresponding to the protons alpha to the coordinating nitrogen of the terminal pyridine rings of the ligand undergo a large downfield shift, attributable to the withdrawal of electron density from the aromatic system following coordination to palladium (added in stoichiometric amounts). A representative ^1^H NMR spectra for cage **C2** formation is shown in Figure S4.

In order to assess whether the synthesized metallacages were soluble in water, the products were suspended in D_2_O (8 mg per mL) and sonicated at 37°C for 24 h. The fine suspension was then filtered, and the filtrate was analyzed by ^1^H NMR. Despite the tetra-cationic nature of the metallacages, and the incorporation of solubilizing entities to the ligand scaffold, none of the metallacages (**C1-C7**) were soluble in D_2_O. In fact, the filtrate solution was colorless and no trace of a proton signal could be found in the ^1^H NMR spectra.

As a second attempt to water solubilization, and taking inspiration from previous work by Altmann and Pöthig (2016) on the synthesis of water soluble Ag(I) and Au(I) pillarplexes, we synthesized new cages featuring acetate as the counterion by the use of palladium(II) acetate as the precursor in the self-assembly. However, this attempt produced an insoluble residue that could not be dissolved in either organic solvents or water.

Concerning cisplatin encapsulation, in the case of previously reported [Pd_2_L_4_]BF_4_ cages (L = (2,6-bis(pyridin-3-ylethynyl)pyridine)) it was hypothesized that their central cavities are lined with four hydrogen bond accepting pyridine units which enable the encapsulation of two cisplatin molecules within the metallosupramolecular architecture through hydrogen bonding interactions between the cage and the amine ligands of the cisplatin guest (Lewis et al., 2012). This idea has been supported by NMR spectroscopy studies (Lewis et al., 2012), where chemical shifts of hydrogens in the trispyridyl ligands were observed upon cisplatin encapsulation. Control experiments confirm the importance of the hydrogen bonding interaction between the cage and the amine ligands of the cisplatin guest. It was also observed that upon addition of small amounts of D_2_O to a solution of the cisplatin-cage host-guest adduct in acetonitrile, the H_a_, and H_b_ proton signals of the cage sharpen and shift upfield again, indicating evacuation of cisplatin from the cavity (Lewis et al., 2012).

**Figure 1:**
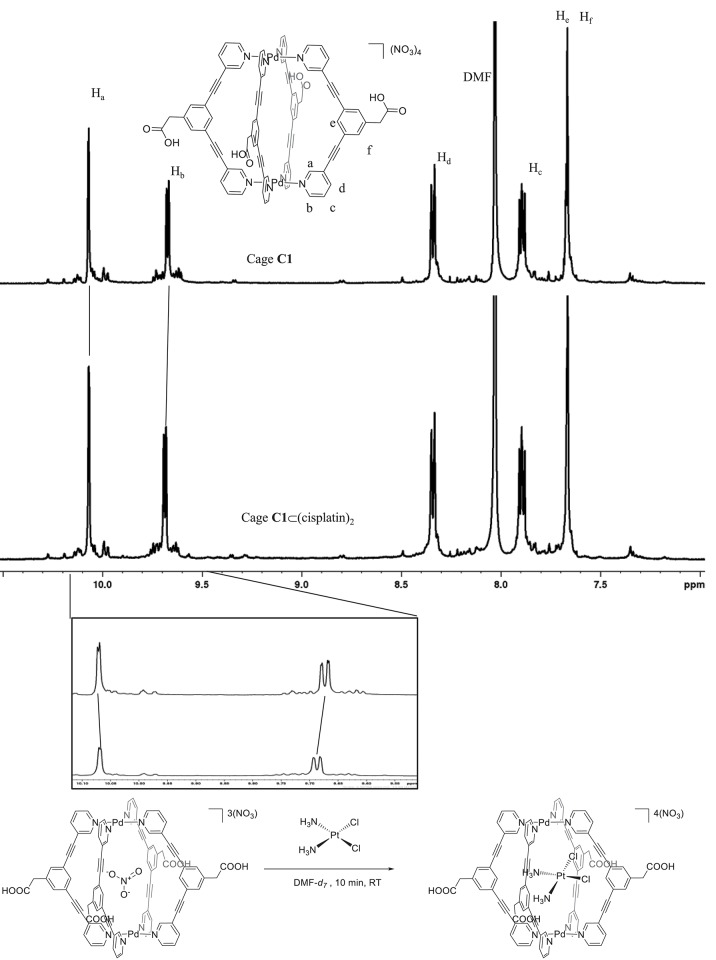
Cisplatin encapsulation in a metallacage following displacement of nitrate ions from the cavity. Stacked spectra of cage **C1** in DMF-*d*_7_ (top) and cage **C1** after the addition of 2 equation of cisplatin (bottom). **Box**: Zoom-in of signals for peaks H_a_ and H_b_ with chemical shifts of δ = −0.002 ppm (upfield) and +0.01 ppm (downfield), respectively.

In this context, information on the encapsulation process of cisplatin within the more hydrophobic cavity of the bispydridyl ligand forming our [Pd_2_L_4_]^4+^ cages, as opposed to the less hydrophobic cavity of trispyridyl-ligand Pd_2_L_4_ metallacages (Lewis et al., 2012, 2014), needs to be achieved still. Thus, we investigated if the encapsulation of the anticancer drug cisplatin could occur in the metallacages using ^1^H NMR spectroscopy. In order to avoid DMSO as the solvent, since it is known to exchange cisplatin's chlorido ligands, the experiment was carried out in DMF-*d*_7_. Cage **C1** [Pd_2_L_4_](NO_3_)_4_ was selected to study the encapsulation process due to its solubility in this solvent. Figure 1 shows the variation of the chemical shifts of selected cage protons upon cisplatin encapsulation. Specifically, we could clearly observe the chemical shifts of peaks H_a_ and H_b_: the peak of H_a_, a cavity-facing proton, showed a small upfield shift of δ −1.0 Hz. The *exo*-facing proton, H_b_, showed a downfield shift upon cisplatin encapsulation, in accordance with previously reported studies (Lewis et al., 2012). Unfortunately, the cavity facing peak H_e_, likely to interact with the guest Pt(II) complex, was masked by the peak of H_f_, and therefore, could not be analyzed.

It is worth mentioning that the observed smaller chemical shifts of the cavity facing proton H_a_, with respect to those reported by Lewis et al. (2012) can be due to the lack of the H-bonding with the cisplatin guest (via N—H_3_N). Moreover, it has been previously reported that ${\text{NO}}_{3}^{-}$ counterions are encapsulated within the cage architecture of trispyridyl Pd_2_L_4_ metallacages featuring a nitrogen in *endo*-position, while ${\text{BF}}_{4}^{-}$ ions are not (Lewis and Crowley, 2014). Thus, we hypothesized that the observed small chemical shift of H_a_ following cisplatin encapsulation was due to the presence of ${\text{NO}}_{3}^{-}$ ions inside the cage, which already induced chemical shifts of H_a_ and H_b_ and thus, acted to “mask” the effect of the cisplatin encapsulation process. In fact, upon evacuation of the anionic ${\text{NO}}_{3}^{-}$ counterion, the “de-shielding” effect of the cavity facing protons from ${\text{NO}}_{3}^{-}$ would be reduced as the neutral guest, cisplatin, occupies the metallacage cavity.

Afterwards, we also investigated if cisplatin would preferentially be encapsulated within our bispyridyl type Pd_2_L_4_ metallacages featuring a different counter ion. Therefore, an analogous metallacage was synthesized with ${\text{BF}}_{4}^{-}$ as the counterion, **C1.BF**_**4**_ and studied for its cisplatin encapsulation properties by ^1^H NMR spectroscopy. Supporting Figure S10 shows the stacked ^1^H NMR spectrum of **C1.BF**_**4**_, alone or encapsulating cisplatin [**C1.BF_4_⊂(cisplatin)**] in DMF-*d*_7_. Indeed, both H_a_ and H_b_ show marked downfield shifts upon cisplatin encapsulation of 0.044 and 0.033 ppm, respectively. In addition, H_e_ also shows a downfield shift of 0.03 ppm. Furthermore, the peaks of both H_a_ and H_b_ of **C1** (before addition of cisplatin) are already shifted further downfield (Figure 1, ca. 0.095 and 0.001 ppm, respectively) when compared to H_a_ and H_b_ of **C1.BF**_**4**_ (Figure S10) consistent with encapsulation of ${\text{NO}}_{3}^{-}$.

To continue our investigation, the effect of the solvent polarity was hypothesized to be the driving force of the cisplatin encapsulation process. Thus, the latter was also tested using **C1.BF**_**4**_ in MeCN-*d*_3_, with reduced polarity with respect to DMF-*d*_7_. However, upon addition of 2 eq. cisplatin to the MeCN-*d*_3_ solution of **C1.BF**_**4**_ (1 equation) a precipitate was formed, even after several hours of sonication. The suspension was filtered, and the resulting solution was analyzed by ^1^H NMR spectroscopy. However, the resulting spectra showed no indication of cisplatin encapsulation (data not shown). Instead, the large upfield shift of the peaks corresponding to the α-protons of the coordinating pyridyl is indicative of the ligands no longer coordinated to Pd(II) ions. Moreover, the NMR spectrum suggests that the structure of the ligand has also been altered.

Therefore, in order to study the effects of the solvent on cisplatin encapsulation, we synthesized a derivative of cage **C1.BF**_**4**_, but where the carboxylic function was protected by a benzyl group, namely cage **C1Bn.BF**_**4**_. Thus, cisplatin encapsulation was followed by ^1^H NMR in MeCN-*d*_3_ and the results are presented in Figure S11. As it can be observed, no chemical shifts could be seen for any of the cage's proton peaks even after 24 h incubation of cisplatin at room temperature. We could conclude that in acetonitrile, cisplatin encapsulation is not favored. In order to check that metallodrug encapsulation was not prevented by the presence of the benzyl group on the cage, we also monitored the process once more in DMF-*d*_7_. The results are depicted in Figure S12 and indicate that, in these conditions, cisplatin encapsulation occurs, as shown by the chemical shifts of H_a_ (downfield, δ = +0.009 ppm), H_b_ (downfield, δ = +0.021 ppm), and a small shift for the cavity-facing proton H_e_ (upfield, δ = −0.002 ppm). The encapsulation of cisplatin in cage **C1Bn.BF**_**4**_ was further studied in DMF-*d*_7_ using ^195^Pt NMR to see if a complementary shift could be observed for the guest cisplatin molecules. Indeed, an upfield shift of −5 ppm was observed upon addition of either 1 eq. or 2 eq. of cisplatin in the presence of metallacage **C1Bn.BF**_**4**_ (Figure S13), supporting cisplatin encapsulation. The obtained results are in agreement with previously reported ones from our group on similar cage systems *exo*-functionalized with different type of ligands (Schmidt et al., 2016c), indicating that surface functionalization does not prevent the guest from entering the cavity.

The encapsulation of cisplatin in **C1Bn.BF**_**4**_ was also studied by ^1^H DOSY NMR in DMF-*d*_7_. Cisplatin alone shows a broad signal at 4.18 ppm, H_NH3_ with a diffusion coefficient = −8.9 × 10^−10^ m^2^/s (Figure S14, red traces). Clear quenching of this signal was observed upon addition of 1 eq. of metallacage **C1Bn.BF**_**4**_ (Figure S14, blue traces). The cisplatin peak reappears slightly upon addition of a second equivalent of cisplatin to **C1Bn.BF_4_⊂(cisplatin)** (Figure S14, black traces), which may suggest that the metallacage cavity has been saturated to form **C1Bn.BF_4_⊂(cisplatin)**_**2**_ and residual cisplatin remains free in solution, or that upon addition of a second equivalent of cisplatin, a dynamic equilibrium occurs.

## Conclusions

In conclusion, we report here on a series of *exo*-functionalized [Pd_2_L_4_]^4+^ metallacages featuring bispydridyl ligands. Despite the addition of hydrophilic groups to the ligands' scaffold, or the use of different counter ions, the increase in water solubility of the resulting cages was not achieved. Thus, different strategies are necessary to address this problem, including the conjugation of the ligands to peptides. The latter approach has already proved to be successful (Han et al., 2017).

Furthermore, the encapsulation of the anticancer drug cisplatin in selected cages has been studied by NMR spectroscopy, and the obtained results show that if the solvent is of sufficient polarity, metallodrug encapsulation can easily occur in the hydrophobic cavity of the cage despite the absence of hydrogen-bond accepting central pyridine units (Lewis and Crowley, 2014). Conversely, polar solvent molecules capable of forming hydrogen-bond networks (including DMF and water) are likely to prefer not to be encapsulated by hydrophobic cage cavities, as in our case, and will not compete with the cisplatin molecules. This hypothesis is corroborated by previous studies on M_4_L_6_ metallacages for which guest encapsulation in polar protic solvents, such as water, appears to be driven by initial desolvation of the guest with concomitant rearrangement of the hydrogen bond networks in solution, more than by host-guest interactions alone (Leung et al., 2008). In general, energetically favorable solvent rearrangement during guest desolvation also explains the ability of the supramolecular host to encapsulate neutral guests such as cisplatin, in spite of the lack of possible electrostatic attraction or H-bond interactions with the charged host, as in our case. Further studies are ongoing in our lab to validate this hypothesis.

## Author Contributions

AC and BW contributed conception and design of the study. BW, MW, and ST contributed to the design, synthesis, and characterization of the ligands, and of the palladium cages. TW contributed to the HR-ESI-MS analysis of the cages. BW and RJ performed the encapsulation studies by NMR spectroscopy. AC and BW wrote the first draft of the manuscript, while MW wrote sections of the manuscript. All authors contributed to manuscript revision, read, and approved the submitted version.

### Conflict of Interest Statement

The authors declare that the research was conducted in the absence of any commercial or financial relationships that could be construed as a potential conflict of interest.
