# Supplementary material for: Exo-Functionalized Metallacages as Host-Guest Systems for the Anticancer Drug Cisplatin
Source: Front Chem. 2019 Feb 18;7:68. doi: 10.3389/fchem.2019.00068 (PMC6387950; doi:10.3389/fchem.2019.00068)
Supplement: Supplementary file 1 [file Table_1.DOCX]

Supplementary Material

*Exo*-Functionalized Metallacages as host-guest systems for the anticancer drug cisplatin

Ben Woods^1^, Margot N. Wenzel^1^, Thomas Williams^1^, Sophie R. Thomas^1^, Robert L. Jenkins,^1^ Angela Casini^1,*^

^1^ School of Chemistry, Cardiff University, Main Building, Park Place, CF10 3AT Cardiff, United Kingdom.

*** Correspondence:**Corresponding Author
[casinia@cardiff.ac.uk](mailto:casinia@cardiff.ac.uk)

# Experimental Section

**General remarks**

*Chemicals.* All reagents and solvents were obtained from commercial suppliers and used without further purification, unless otherwise stated. Triethylamine was distilled under nitrogen before use. ^1^H-NMR, ^13^C{^1^H}-NMR, ^11^B-NMR and ^19^F-NMR spectra were recorded on a 500 MHz DMX (Bruker) or 400 MHz AV spectrometer (Bruker). Chemical shifts are given in parts per million (ppm). Abbreviations for NMR multiplicities are: singlet (s), doublet (d), triplet (t), multiplet (m), broad (b). Coupling constants J are given in Hz. The following solvents were used as internal standards: DMSO*-d*_6_: 2.50 ppm (^1^H-NMR) and 39.52 ppm (^13^C-NMR); CDCl_3_: 7.26 ppm (^1^H-NMR) and 77.16 ppm (^13^C-NMR).^1^ High resolution ESI-MS spectra were recorded on a Walter Synapt G2SI QTOF. IR spectra were recorded on a Shimadzu IRAffinity-1S FT IR spectrophotometer.

Column chromatography was carried out using a Biotage Isolera^TM^ Prime automated purification system, with ZIP KP 10 g silica gel cartridges, using the following solvent gradients. Solvent A: Hexane; solvent B: ethylacetate; solvent C: Methanol. Gradient A: A/B (0% - 100%, 30 column volumes (CV)), B/C (0 – 10%, 10 CV), B/C (10%, 10 CV). Gradient B: A/B (20% - 30%, 6 CV), A/B (30% - 75%, 15 CV), A/B (75%, 5 CV), A/B (75% - 100%, 5 CV), B/C (0% - 10%, 10 CV).

**Synthesis**

Synthesis of ligands **L1** and **L2**

Ligands **L1** and **L2** were synthesized according to scheme **S1**.

**Scheme S1.** Scheme of the synthesis of ligands **L1** and **L2**. Benzyl protection of the acid group to form the benzyl protected precursor **BnP1** and **BnP2**, followed by Sonogashira cross-coupling to afford the benzyl protected ligand **BnL1** and **BnL2**. Deprotection of the group is achieved under basic conditions to give the ligands **L1** and **L2**.

Ligand 3,5-bis(3-ethynylpyridine)-phenylacetic acid (**L1**)

**Benzyl-(3,5-dibromophenylacetate)** (**BnP1**) was synthesized according to a previously reported procedure.^2^

# Benzyl-(3,5-bis(3-ethynylpyridine)-phenylacetate) (BnL1): A mixture of benzyl-(3,5-dibromophenylacetate, BnP1) (370 mg, 1.0 mmol, 1.0 eq.), 3-ethynylpyridine (309 mg, 3.0 mmol, 3.0 eq.), Pd(PPh_3_)_2_Cl_2_ (68.1 mg, 0.1 mmol, 0.1 eq.) and CuI (18.5 mg, 0.1 mmol, 0.1 eq.), was suspended in distilled triethylamine (15 mL) and stirred under a nitrogen atmosphere at 90°C. After 24 h, the reaction mixture was diluted with ethylacetate (50 mL) and filtered over glass-fritted funnel (por. 3). The solvent was removed under vacuum and the crude residue further purified by column chromatography (gradient A) to give the product benzyl-(3,5-bis(3-ethynylpyridine)-phenylacetate) (BnL1) as an off white solid (311 mg, 0.8 mmol, 75%).

# ^1^H NMR (400 MHz, DMSO*-d*_6_): δ [ppm] = 8.80 (d, J = 1.4 Hz, 2H, H_a_), 8.61 (dd, J = 1.6, 5.0 Hz, 2H, H_b_), 8.12 (d, J = 1.6 Hz, 2H, H_f_), 8.06 (t, J = 1.6, 1H H_e_), 8.02 (dt, J = 1.8, 8.0 Hz, 2H, H_d_) 7.64-7.35 (m, 7H, phenyl + H_c_), 5.39 (s, 2H, H_g_).

**^13^C{^1^H} NMR** (101 MHz, DMSO*-d*_6_): δ [ppm] = 164.5, 152.3, 149.9, 139.3, 138.7, 136.1, 132.5, 131.9, 131.4, 129.2, 129.1, 128.8, 128.7, 124.1, 123.3, 119.2, 90.5, 88.6, 67.4.

**ESI-MS** calcd. for C_28_H_19_N_2_O_2_ [M+H]^+^: *m/z* = 415.1447; found: 415.1448; δ = 0.2 ppm.

**Benzyl-(3,5-bis(3-ethynylpyridine)-phenylacetate)** (**L1**): **BnL1** (768 mg, 1.8 mmol, 1 eq.) was dissolved in acetonitrile (50 mL). NaOH (211 mg, 5.4 mmol, 3.0 eq.) was dissolved in water (7 mL) and added to the organic solution. The reaction was stirred at 90°C for 5 h. The solution was cooled to room temperature and the solution was concentrated in vacuo (approx. 5 mL). The concentrated solution was acidified drop wise with concentrated HCl (~pH 5) and the resultant precipitate was collected by filtration. The solid was washed with cold ethyl acetate and diethyl ether to give the product as an off-white solid (442 mg, 1.3 mmol, 73%).

**^1^H NMR** (400 MHz, CDCl_3_): δ [ppm] 8.80 (s, 2H, H_a_), 8.56 (d, J = 3.4 Hz, 2H, H_b_), 7.83 (dt, J = 1.7 Hz, 8.0 Hz, 2H, H_d_), 7.65 (t, J = 1.4 Hz, 1H, H_e_), 7.55 (d, J = 1.3 Hz, 2H, H_f_), 7.33 (dd, J = 4.7 Hz, 8.2 Hz, 2H, H_c_), 3.70 (s, 2H, H_g_).

**^13^C{^1^H} NMR** (101 MHz, DMSO*-d*_6_): δ [ppm] 172.6 (C_COOH_), 152.2 (C_a_), 149.7 (C_b_), 139.2 (C_e_), 133.7, 132.7, 124.2, 122.7, 119.6, 91.6 (C_Alkyne_), 87.4 (C_Alkyne_).

**ESI-MS** calcd. for C_22_H_14_N_2_O_2_ [M+H]^+^: *m/z* = 339.1134; found 339.1148; δ = 4.1 ppm.

Ligand 3-(3,5-Bis(3-ethynylpyridine)phenyl)-propionic acid (**L2**)

**Benzyl-(3-(3,5-dibromophenyl)propanoate) (BnP2)** was synthesized according to a previously reported procedure.^2^

**Benzyl-(3-(3,5-bis(3-ethynylpyridine)phenyl)-propanoate)** **(BnL2):** A mixture of benzyl-(3(3,5-dibromophenyl)propanoate) (365 mg, 0.9 mmol, 1.0 eq.), 3-ethynylpyridine (284 mg, 2.8 mmol, 3.0 eq.), Pd(PPh_3_)_2_Cl_2_ (64 mg, 0.1 mmol, 0.1 eq.), and CuI (19 mg, 0.1 mmol, 0.1 eq.), was suspended in distilled triethylamine (15 mL) and stirred under a nitrogen atmosphere at 90°C. After 24 h, the reaction mixture was diluted with ethyl acetate (50 mL) and filtered over glass-fritted funnel (por. 3). The solvent was removed in vacuo and the crude residue further purified by column chromatography (gradient A) to give the product benzyl-(3-(3,5-bis(3-ethynylpyridine)phenyl)propanoate) (**BnL2**) as an orange oil (236 mg, 0.5 mmol, 58%).

**^1^H NMR** (400 MHz, DMSO*-d*_6_): δ [ppm] = 8.80 (d, J = 1.4 Hz, 2H, H_a_), 8.61 (dd, J = 1.6, 5.0 Hz, 2H, H_b_), 8.12 (d, J = 1.6 Hz, 2H, H_f_), 8.06 (t, J = 1.6 Hz, 1H, H_e_), 8.02 (dt, J = 1.8, 8.0 Hz, 2H, H_d_), 7.64-7.35 (m, 7H, phenyl, H_c_), 5.39 (s, 2H, H_g_).

**^13^C{^1^H} NMR** (101 MHz, DMSO*-d*_6_): δ [ppm] = 164.5 (C_COOH_), 152.3 (C_a_), 149.9 (C_b_), 139.3 (C_e_), 138.7 (C_d_), 136.1, 132.5, 131.9 (C_phenyl_), 131.4 (C_phenyl_), 129.2 (C_phenyl_), 129.1 (C_phenyl_), 128.8 (C_phenyl_), 128.7 (C_phenyl_), 124.1, 123.3, 119.2, 90.5 (C_Alkyne_), 88.6 (C_Alkyne_), 67.4.

**ESI-MS** calcd. for C_28_H_19_N_2_O_2_ [M+H]^+^: *m/z* = 415.1447; found: 415.1448; δ = 0.2 ppm.

**3-(3,5-Bis(3-ethynylpyridine)phenyl)-propionic acid (L2): BnL2** (481 mg, 1.1 mmol, 1.0 eq.) was dissolved in acetonitrile (20 mL). NaOH (108 mg, 2.7 mmol, ~2.5 eq.) was dissolved in water (4 mL) and added to the organic solution. The reaction was stirred at 90°C for 5 h. The solution was cooled to room temperature and the solution was concentrated in vacuo (approx. 5 mL). The concentrated solution was acidified drop wise with conc. HCl (~pH 5) and the resultant precipitate was collected by filtration. The solid was washed with cold ethyl acetate and diethyl ether to give the product **L2** as an off-white solid (293 mg, 0.83 mmol, 75%).

**^1^H NMR** (400 MHz, CDCl_3_): δ [ppm] 12.28 (s, 1H, H_i_), 8.84 (d, J = 2.1 Hz, 2H, H_a_), 8.67 (dd, J = 1.4 Hz, 4.8 Hz, 2H, H_b_), 8.06 (dt, J = 1.7 Hz, 8.0 Hz, 2H, H_d_), 7.70 (s, 1H, H_e_), 7.61 (d, J = 0.9, 2H, H_f_), 7.54 (dd, J = 4.9 Hz, 7.8 Hz, 2H, H_c_), 2.93 (t, J = 7.4, 2H, H_h_), 2.68 (t, J = 7.7, 2H, H_g_).

**^13^C{^1^H} NMR** (101 MHz, DMSO*-d*_6_): δ [ppm] 173.6 (C_COOH_), 151.7 (C_a_), 149.2 (C_b_), 142.6, 138.7, 132.1, 132.0, 123.7, 122.4, 119.1, 91.3 (C_Alkyne_), 86.8 (C_Alkyne_), 34.6 (C_h_), 29.7 (C_g_).

**ESI-MS** calcd. for C_23_H_17_N_2_O_2_ [M+H]^+^: *m/z* = 353.1290; found 353.1289; δ = -0.3 ppm.

3,5-bis(3-ethynylpyridine)-1-(2-(2-methoxyethoxy)ethane)phenol **(L3)**

Ligand **L3** was synthesized according to the scheme **S2**.

**Scheme S2**. Scheme of the synthesis of ligand **L3**. Sonogashira cross coupling between 3,5-dibromophenol and 3-ethynylpyridine gave precursor ligand **P3**. This was then coupled to 1-chloro-2-(2-methoxyethoxy)ethane to produce 3,5-bis(3-ethynylpyridine)-1-(2-(2-methoxyethoxy)ethane)phenol (**L3**).

**3,5-bis(3-ethynylpyridine)phenol (P3)**: 3,5-dibromophenol (1.00 g, 4.0 mmol, 1.0 eq.), 3-ethynylpyridine (1.22 g, 11.9 mmol, 3.0 eq.), CuI (76 mg, 0.4 mmol, 0.1 eq.), Pd(PPh_3_)_2_Cl_2_ (278 mg, 0.4 mmol, 0.1 eq.) were added to a flame dried round bottom flask under nitrogen. Triethylamine (40 mL) was added and the suspension was stirred at 90°C for 72 hours. Ethylacetate (100 mL) was added and the reaction mixture was filtered over Celite. The filtrate was concentrated *in vacuo.* The residue was dissolved in DCM and loaded onto silica gel before purification via chromatography (gradient A) to give the product as a white solid (800 mg, 2.7 mmol, 68% yield).

**^1^H NMR** (400 MHz, DMSO*-d*_6_): δ [ppm] 10.20 (b, 1H, H_O_*_H_*), 8.77 (dd, J = 0.8 Hz, 2.2 Hz, 2H, H_a_), 8.60 (dd, J = 1.7 Hz, 5.0 Hz, 2H, H_b_), 7.99 (dt, J = 1.9 Hz, 7.8 Hz, 2H, H_d_), 7.48 (ddd, J = 0.8 Hz, 4.9 Hz, 7.9 Hz, 2H, H_c_), 7.24 (t, J = 1.1 Hz, 1H, H_e_), 7.02 (d, J = 1.6 Hz, 2H, H_f_).

**^13^C{^1^H} NMR** (101 MHz, DMSO*-d*_6_): δ [ppm] 152.2 (C_a_), 149.7 (C_b_), 139.2, 125.7, 124.1, 123.8, 119.6, 119.4, 110.2, 91.8 (C_alkyne_), 86.9 (C_alkyne_).

**ESI-MS** calcd. for C_20_H_13_N_2_O [M+H]^+^: *m/z* = 297.1028; found 297.1162; δ = 45 ppm.

**3,3'-((5-(2-(2-methoxyethoxy)ethoxy)-1,3-phenylene)bis(ethyne-2,1-diyl))dipyridine (L3)**: 3,5-bis(3-ethynylpyridine)-phenol (51 mg, 0.3 mmol, 1.0 eq.), and K_2_CO_3_ (46 mg, 0.6 mmol, 2 eq.) were dissolved in dry DMF (5 mL) and added to a flame dried RBF. 1-chloro-2-(2-methoxyethoxy)ethane (synthesized according to a previously reported procedure)^3^ (61 mg, 0.4 mmol, 1.5 eq.) was added to this solution and the reaction was stirred at 90°C for 16 h under nitrogen. The reaction mixture was filtered over Celite and the solvent was removed *in vacuo*. The residue was taken up in DCM (50 mL) and washed with distilled water (3 x 30 mL) and brine (1 x 30 mL). The organic phase was dried over MgSO_4_ and the solvent removed *in vacuo*. The crude residue was purified by chromatography (gradient A) to give the product as a yellow oil (366 mg, 0.17 mmol, 58%).

**^1^H NMR** (400 MHz, MeOD*-d*_4_): δ [ppm] 8.68 (s, 2H, H_a_), 8.50 (d, J = 4.33 Hz, 2H, H_b_), 7.94 (dt, J = 2.0 Hz, 2H, H_d_), 7.43 (dd, J = 4.8, 8.0 Hz, 2H, H_d_), 7.30 (s, 1H, H_e_), 7.14 (s, 2H, H_f_), 4.16 (t, J = 4.8 Hz, 2H, H_g_), 3.83 (t, J = 4.4 Hz, 2H, H_h_), 3.71-3.67 (m, 2H, H_i_*),* 3.58-3.54 (m, 2H, H_j_), 3.34 (s, 3H, H_k_).

**^13^C{^1^H} NMR** (101 MHz, MeOD*-d*_4_): δ [ppm] 158.8, 151.2, 148.1, 139.2, 127.0, 123.8, 123.7, 120.5, 118.2, 91.4 (C_alkyne_), 85.6 (C_alkyne_), 71.6 (C_g_), 70.2 (C_h_), 69.3 (C_i_), 67.7 (C_j_), 57.9 (C_k_).

**ESI-MS** calcd. for C_25_H_23_N_2_O_3_ [M+H]^+^: *m/z* = 399.1709; found 399.1799; δ = 22.5 ppm.

Synthesis of ligands **L4** and **L5**

The synthesis of ligands **L4** and **L5** was achieved according to scheme **S3**.

**Scheme S3.** Route of the synthesis of ligands **L4** and **L5**.

**3,5-bis(3-ethynylpyridine)-benzylalcohol (P4):** 3,5-dibromobenzylalcohol (1.00 g, 3.8 mmol, 1.0 eq.), 3-ethynylpyridine (1.16 g, 11.3 mmol, 3 eq.), CuI (72 mg, 0.4 mmol, 0.1 eq.), Pd(PPh_3_)_2_Cl_2_ (264 mg, 0.4 mmol, 0.1 eq.) were added to a flame dried flask. Triethylamine (40 mL) was added and the suspension was stirred under nitrogen at 90°C for 24 h. Ethylacetate (100 mL) was added and the reaction mixture was filtered over Celite. The filtrate was concentrated *in vacuo*. The residue was dissolved in DCM and loaded onto silica gel before purification *via* chromatography (gradient A) to give the product as a white solid (828 mg, 2.67 mmol, 71% yield).

**^1^H NMR** (400 MHz, DMSO*-d*_6_): δ [ppm] 8.79 (d, J = 1.9 Hz, 2H, H_a_), 8.62 (dd, J = 1.3 Hz, 4.8 Hz, 2H, H_b_), 8.02 (dt, J = 1.9 Hz, 8.0 Hz, 2H, H_d_) 7.69 (s, 1H, H_e_), 7.61 (s, 2H, H_f_), 7.50 (dd, J = 4.9 Hz, 8.0 Hz, 2H, H_c_), 5.44 (t, J = 5.7 Hz, 1H, OH), 4.57 (d, J = 5.8, 2H, H_g_).

**^13^C{^1^H} NMR** (101 MHz, DMSO*-d*_6_): δ [ppm] 152.2 (C_a_), 149.7 (C_b_), 144.8, 139.2, 130.3, 124.2, 122.7, 119.6, 91.7 (C_alkynyl_), 87.2 (C_alkynyl_), 62.3, 31.2.

**3,5-bis(3-ethynylpyridine)-4-benzylbromide (P5):** 3,5-bis(3-ethynylpyridine)-4-benzylalcohol (200 mg, 0.6 mmol, 1.0 eq.), carbon tetrabromide (320 mg, 0.9 mmol, 1.5 eq.) and triphenylphosphine (200 mg, 0.7 mmol, 1.2 eq.) were added to a flame dried flask. Dry DCM (10 mL) was added and the solution was stirred in the absence of light, at room temperature, under nitrogen, for 4 h. The solution was loaded directly onto silica gel and purified *via* chromatography (gradient B) to give the product as a white solid (133 mg, 0.36 mmol, 56%).

**^1^H NMR** (400 MHz, CDCl_3_): δ [ppm] 8.76 (d, J = 2.0 Hz, 2H, H_a_), 8.57 (dd, J = 1.8 Hz, 4.8 Hz, 2H, H_b_), 7.80 (dt, J = 1.9 Hz, 7.9 Hz, 2H, H_d_), 7.65 (t, J = 1.4 Hz, 1H, H_e_), 7.56 (d, J = 1.4 Hz, 2H, H_f_), 7.30 (ddd, J = 0.8 Hz, 4.9 Hz, 7.9 Hz, 2H, H_c_), 4.45 (s, 2H, H_g_).

**^13^C{^1^H} NMR** (101 MHz, DMSO*-d*_6_): δ [ppm] 152.2 (C_a_), 149.9 (C_b_), 143.4, 139.3 (C_e_), 134.5, 133.1, 224.2, 123.4, 119.4, 91.1 (C_alkynyl_), 88.1(C_alkynyl_), 32.7 (C_g_).

**3,5-bis(3-ethynylpyridine)-4-benzyl(thio-β-D-glucose tetraacetate) (L4)**: 1-thio-β-D-glucose tetraacetate (104 mg, 0.3 mmol, 1.1 eq.) and bis-3,5-(3-ethynylpyridine)-4-benzylbromide (97 mg, 0.3 mmol, 1.0 eq.) was dissolved in acetonitrile (6 mL) in a flame dried flask. Triethylamine (40 µL, 0.3 mmol, 1.1 eq.) was added and the solution was stirred for 3 h at reflux before being quenched with water. The reaction mixture was extracted with ethyl acetate (3 x 50 mL). The organic layer was dried over MgSO_4_ and concentrated in vacuo. The residue was purified *via* chromatography (gradient A) to give the product as a yellow solid (160 mg, 0.22 mmol, 84% yield).

**^1^H NMR** (400 MHz, CDCl_3_): δ [ppm] 8.75 (dd, J = 0.8 Hz, 2.1 Hz, 2H, H_a_), 8.55 (dd, J = 1.7 Hz, 4.9 Hz, 2H, H_b_), 7.80 (dt, J = 1.9 Hz, 7.9 Hz, 2H, H_d_), 7.63 (t, J = 1.5 Hz, 1H, H_e_), 7.48 (d, J = 1.5 Hz, 2H, H_f_), 7.29 (ddd, J = 1.0 Hz, 4.9, 7.9 Hz, 2H, H_c_) 5.16 (d, J = 9.3 Hz, 1H, H_h_), 5.11-5.04 (m, 2H, H_g_), 4.35 (d, J = 10.0 Hz, 1H, H_l_) 4.27-4.04 (m, 3H, H_i_, H_j_, H_k_), 3.88 (m, 2H, H_m_, H_m’_), 3.68-3.62 (m, 1H, H_l_).

**^13^C{^1^H} NMR** (101 MHz, CDCl_3_): δ [ppm] = 169.6 (C_Ac_), 169.1 (C_Ac_), 168.4 (C_Ac_), 168.4 (C_Ac_), 151.3 (C_a_), 147.9 (C_b_), 137.5, 137.1, 132.7, 131.3, 122.4, 122.1, 118.9, 90.2 (C_alkynyl_), 86.0 (C_alkynyl_), 81.0, 74.9, 72.7, 68.7, 67.2, 61.1, 32.0 (C_g_), 19.7 (C_Ac_), 19.7 (C_Ac_), 19.6 (C_Ac_), 19.6 (C_Ac_).

**ESI-MS** calcd. for C_35_H_32_N_2_O_9_S [M+H]^+^: *m/z* = 657.1907; found 657.1916; δ = 1.4 ppm.

**3,5-bis(3-ethynylpyridine)-4-benzyl-thio-β-D-glucose (L5): L4** (114 mg, 0.2 mmol, 1.0 equiv.) was dissolved in methanol (10 mL). To this solution was added Amberlite 401 OH- ion exchange beads (200 mg) and the reaction was stirred at room temperature for overnight. The Amberlite beads were removed by filtration and the organic solution was concentrated *in vacuo*. Water (30 mL) was added to the colourless residue, and the resulting suspension was filtered to afford the product as a white solid (42 mg, 87 µmol, 51% yield).

**^1^H NMR** (400 MHz, MeOD*-d*_4_): δ [ppm] 8.73 (dd, J = 0.9 Hz, 2.2 Hz, 2H, H_a_), 8.54 (dd, J = 1.7 Hz, 5.0 Hz, 2H, H_b_), 8.01 (dt, J = 2.0 Hz, 8.2 Hz, 2H, H_d_), 7.67-7.63 (m, 3H, H_e_, H_f_), 7.48 (ddd, J = 0.9 Hz, 4.9 Hz, 8.0 Hz, 2H, H_c_), 4.23-4.05 (m, 2H), 3.96-3.86 (m, 2H), 3.74-3.67 (m, 1H), 3.29-3.21 (m, 4H).

**^13^C{^1^H} NMR** (101 MHz, MeOD*-d*_4_): δ [ppm] = 151.2, 148.1, 139.2, 132.6, 123.7, 123.0, 120.5, 91.3, 85.7, 80.8, 73.0, 70.2, 61.6, 56.9, 56.0, 54.9.

**ESI-MS** calcd. for C_27_H_25_N_2_O_5_S [M+H]^+^: *m/z* = 489.1484; found 489.1486; δ = 0.4 ppm.

Synthesis of ligands **L6** and **L7**

Ligands **L6** and **L7** were synthesized according to scheme **S4**.

Scheme **S4**. Synthetic route of ligands **L6** and **L7**.

**3,5-bis(3-ethynylpyridine)-4-benzylazide (P6):** 3,5-bis(3-ethynylpyridine)-4-benzylbromide **P5** (106 mg, 0.3 mmol, 1.0 eq.), and sodium azide (18 mg, 0.3 mmol, 1.0 eq.) were stirred in DMF (5 mL) at room temperature overnight. Excess water was added to quench to reaction. The resulting suspension was filtered, and the precipitate was taken up in 1:3 isopropanol/DCM (50 mL), dried over MgSO_4_, filtered and the solvent removed *in vacuo*. The product was isolated *via* chromatography (gradient A) and the product was obtained as an off-white solid (74 mg, 0.2 mmol, 79%).

**^1^H NMR** (400 MHz, CDCl_3_): δ [ppm] 8.77 (s, 2H, H_a_), 8.57 (d, J = 3.6 Hz, 2H, H_b_), 7.80 (dt, J = 1.8 Hz, 7.9 Hz, 2H, H_d_), 7.69 (t, J = 1.4 Hz, 1H, H_e_), 7.48 (s, 2H, H_f_), 7.29 (dd, J = 4.9 Hz, 7.8 Hz, 2H, H_c_), 4.38 (s, 2H, H_g_).

**^13^C{^1^H} NMR** (101 MHz, CDCl_3_): δ [ppm] 171.3, 152.2, 149.8, 139.1, 136.5, 133.7, 133.1, 132.5, 132.0, 131.9, 91.4, 87.5, 66.5.

**ESI-MS** calc. for C_21_H_14_N_5_ [M+H]^+^: *m/z* = 336.1249; found 336.1244; δ = -1.5 ppm.

**2-(acetoxymethyl)-6-((1-(3,5-bis(pyridin-3-ylethynyl)benzyl)-1H-1,2,3-triazol-4-yl)methoxy) tetra-hydro-2H-pyran-3,4,5-triyl triacetate (L6):** The synthesis of ligand **L6** was adapted from a previously reported procedure.^4^ In details, 3,5-bis(3-ethynylpyridine)-4-benzylazide **P6** (100 mg, 0.3 mmol, 1.0 eq.) was dissolved in a solution of 3:1 methanol:water (40 mL). Ascorbic acid (71 mg, 0.4 mmol, 1.3 eq.) and copper sulphate pentahydrate (7 mg, 0.03 mmol, 0.1 equiv.) were dissolved in distilled water (2.5 mL). Saturated NaOH aqueous solution (0.5 mL) was added to the aqueous copper sulphate solution and stirred for 10 min. The two solutions were combined before 2-propyl-tetra-O-acetyl-β-D-glucopyranide was added and the resulting solution was left to stir at room temperature overnight. The reaction was monitored by TLC (1:1 Hex:EtOAc, R_f_ = 0.73) to ensure completion. Saturated NH_4_.OAc_(aq)_ (50 mL) was added to precipitate the product. The aqueous suspension was washed with ethyl acetate (4 x 50 mL). The combined organic phases were washed with brine and dried over MgSO_4_. The solvent was removed *in vacuo* to give the product as an off white solid (182 mg, 0.25 mmol, 84%).

**^1^H NMR** (400 MHz, DMSO*-d*_6_): δ [ppm] 10.12 (s, 1H, H_h_), 8.77 (d, J = 1.9 Hz, 2H, H_a_), 8.60 (dd, J = 1.4, 4.9 Hz, 2H, H_b_), 8.00 (dt, J = 1.7, 7.7 Hz, 2H, H_d_), 7.48 (dd, J = 4.9, 7.9 Hz, 2H, H_c_), 7.25 (t, J = 1.2 Hz, 1H, H_e_), 7.02 (d, J = 1.3 Hz, 2H, H_f_), 5.30 (t, J = 9.5, 1H, H_k­_), 4.96-4.87 (m, XH), 4.77 (dd, J = 8.0, 9.6 Hz, 2H, H_i_) 4.38 (d, J = 2.5 Hz, 1H), 4.34 (d, J = 2.5 Hz, 2H), 4.27 (d, J = 2.6 Hz, 2H), 4.23 (d, J = 2.5 Hz, 1H), 4.22-4.25 (m, 2H), 2.02 (s, 3H, H_OAc_), 2.00 (s, 3H, H_OAc_), 1.98 (s, 3H, H_OAc_), 1.94 (s, 3H, H_OAc_).

**^13^C{^1^H} NMR** (101 MHz, DMSO*-d*_6_): δ [ppm] 170.1 (C_OAc_), 169.5 (C_OAc_), 169.3 (C_OAc_), 169.0 (C_OAc_), 157.7, 151.8, 149.2, 138.7, 131.0, 125.4, 123.4, 119.0, 97.8, 91.3, 86.5, 79.2, 77.9, 72.0, 70.8, 70.6, 68.1, 61.6, 55.9, 20.5 (C_OAc_), 20.4 (C_OAc_), 20.4 (C_OAc_), 20.3 (C_OAc_).

**ESI-MS** calc. for C_36_H_33_N_5_O_8_ [M-(CH_3_COO)]^+^: *m/z* = 663.2329; found 663.4550.

**2-((1-(3,5-bis(pyridin-3-ylethynyl)benzyl)-1H-1,2,3-triazol-4-yl)methoxy)-6-(hydroxymethyl)tetrahydro-2H-pyran-3,4,5-triol (L7):** **L6** (135 mg, 0.18 mmol, 1 eq.) was dissolved in methanol (30 mL). To this solution was added Amberlite IRA 401 (OH^-^) ion exchange beads (230 mg) and the reaction was stirred at room temperature for 16 h. The Amberlite beads were removed by filtration and the organic solution was concentrated *in vacuo*. Water (50 mL) was added to the colourless residue, and the resulting precipitate was filtered to afford the product as a white solid (61 mg, 0.11 mmol, 63%).

**^1^H NMR** (400 MHz, DMSO*-d*_6_/MeOD*-d*_4_): δ [ppm] 8.79 (s, 2H, H_a_), 8.62 (s, 2H, H_b_), 8.28 (s, 1H, H_h_), 8.05-7.98 (m, 2H, H_d_), 7.80 (s, 1H, H_e_), 7.68 (d, J = 1.2 Hz, 1H, H_f_), 7.63 (d, J = 1.3 Hz, 1H, H_f’_), 7.53-7.46 (m, 2H, H_c_), 5.68 (s, 2H, H_g_), 4.87 (d, J = 12.5 Hz, 1H, H_i_), 4.66 (d, J = 12.5 Hz, 1H, H_i’_), 3.72 (d, J = 11.8 Hz, 2H, H_j_) 3.25-2.95 (m, 5H, H_Glucose_).

**^13^C{^1^H} NMR** (101 MHz, CDCl_3_): δ [ppm] 152.2, 149.9, 144.8, 139.2, 138.1, 134.2, 132.0, 125.1, 124.2, 123.3, 119.4, 102.7, 91.1, 87.9, 77.4, 77.2, 73.8, 70.6, 62.0, 61.6, 52.9, 52.3.

**ESI-MS** calc. for C_30_H_28_N_5_O_7_ [M+H]^+^: *m/z* = 554.2039; found 554.3144.

General procedure for the synthesis of metallacages **C1**-**C7**

Synthesis of the cages was achieved by self-assembly (Scheme S5). A solution of the palladium(II) nitrate dihydrate (2 eq.) and ligand (4 eq.) in DMSO was stirred at room temperature for 1 h. After precipitation by addition of acetone and diethylether, the solid was filtered and washed with cold acetone and diethyl ether to yield the cage compound as an off-white solid.

**Scheme S5.** General scheme of the synthesis of Pd_2_L_4_ metallacages **C1-C7** by self-assembly from ligands **L1-L7**.

**Cage C1**: 3,5-bis(3-ethynylpyridine)-phenylacetic acid (**L1**) (34 mg, 0.1 mmol, 4 eq.) and palladium(II) nitrate dihydrate (13 mg, 0.05 mmol, 2 eq.) or tetrakis-aceetonitrile-palladium(II) tetrafluoroborate (22 mg, 0.05 mmol, 2 eq.) were added to DMSO (3 mL) to form cage **C1.NO_3_** (34 mg, 19 µmol, 76%) or **C1.BF_4_** (32 mg, 17 µmol, 66%), respectively.

**C1.NO_3_: ^1^H NMR** (400 MHz, DMSO-*d*_6_): δ [ppm] 9.74 (s, 2H, H_a_), 9.40 (d, J = 5.5 Hz, 2H, H_b_), 8.26 (d, J = 7.9 Hz, 2H, H_d_), 7.91-7.72 (m, 3H, H_c_ + H_e_), 7.63 (s, 2H, H_f_), 3.68 (s, 2H, H_g_).

**^13^C{^1^H} NMR** (101 MHz, DMSO*-d*_6_): δ [ppm] 172.4, 153.4, 151.0, 143.2, 137.6, 134.9, 127.8, 122.7, 122.0, 94.1, 85.5.

**ESI-MS** calc. for C_88_H_60_N_8_O_4_Pd_2_ [M-4O+4H]^2+^: *m/*z = 1506.2847; found 1506.3604.

**IR** (ATR): [cm^-1^] 544, 687, 808, 872, 949, 1015, 1109, 1188, 1227, 1323, 1418, 1481, 1589, 1717, 2220, 2367, 2916, 3071.

**
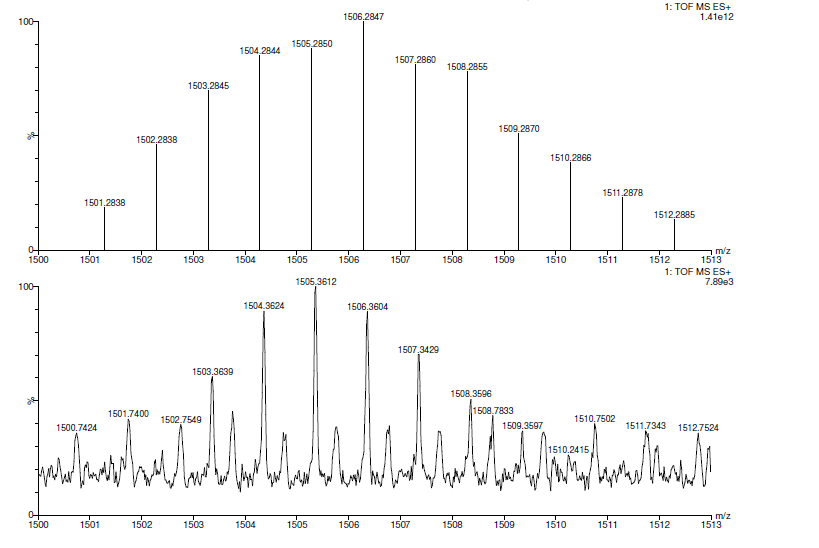
**

**Figure S1**. Comparison between the theoretical isotopic pattern (top) and the experimental pattern (bottom) of [**C1**-4OH]^2+^.

**C1.BF_4_**: **^1^H NMR** (400 MHz, DMSO*-d*_6_): δ [ppm] 9.65 (s, 8H, H_a_), 9.38 (d, J = 5.4 Hz, 8H, H_b_), 8.27 (d, J = 7.25 Hz, 8H, H_d_), 7.88-7.77 (m, 12H, H_e_, H_c_), 7.63 (s, 8H, H_f_), 3.68 (s, 8H, H_g_).

**^11^B{^1^H} NMR** (128 MHz, DMSO*-d*_6_): δ [ppm] -1.33 (BF_4_).

**^19^F{^1^H} NMR** (376 MHz, DMSO*-d*_6_): δ [ppm] 148.2 (BF_4_).

**ESI-MS** calc. for C_88_H_56_N_8_O_8_Pd_2_B_2_F_8_ [M-2H-2BF_4_]^2+^: *m/z =* 870.1202; found: *m/z* = 870.1283.


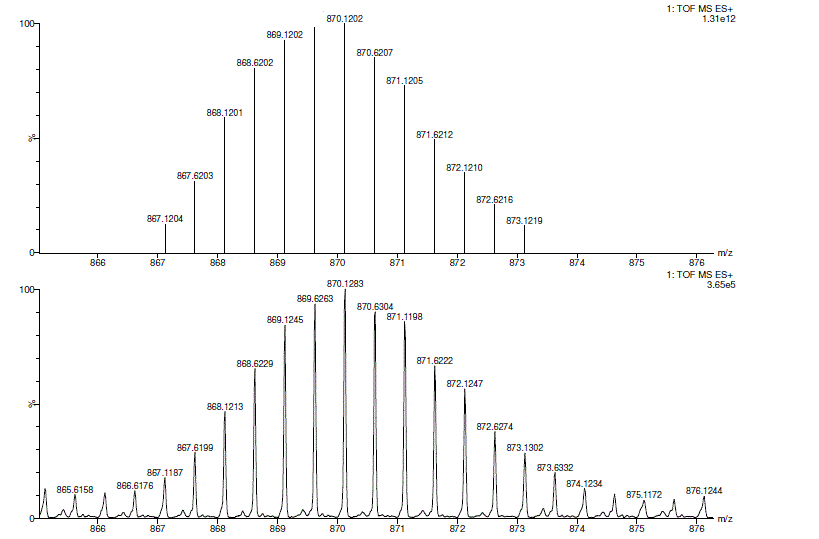
**IR** (ATR): [cm^-1^] 413, 544, 631, 687, 808, 870, 916, 949, 1015, 1188, 1227, 1323, 1414, 1481, 1589, 1709, 2218, 2361, 2913, 3071.

**Figure S2.** Comparison between the theoretical isotopic pattern (top) and the experimental pattern (bottom) of [**C1.BF_4_**-2H-2BF_4_]^2+^.

**Cage BnC1.BF_4_**: Benzyl-(3,5-bis(3-ethynylpyridine)-phenylacetate) **(BnL1)** (43 mg, 0.1 mmol, 4 eq.) and tetrakisaceetonitrilepalladium(II) tetrafluoroborate (22 mg, 0.05 mmol, 2 eq.) were added to DMSO (5 mL) to form cage **BnC1.BF_4_** (49 mg, 22 µmol, 87%).

**^1^H NMR** (400 MHz, DMSO*-d*_6_): δ [ppm] 9.60 (s, 8H, H_a_), 9.37 (d, J = 5.9 Hz, 8H, H_b_), 8.26 (d, J = 8.0 Hz, 8H, H_d_), 7.88-7.79 (m, 12H, H_c_ + H_e_), 7.66 (d, J = 1.3 Hz, 8H, H_f_), 7.33-7.24 (m, 20H, H_phenyl_, 5.09 (s, 8H, H_h_), 3.87 (s, 8H, H_g_).

**^13^C{^1^H} NMR** (101 MHz, DMSO*-d*_6_): δ [ppm] 170.9, 153.3, 151.2, 145.7, 143.3, 137.0, 136.4, 135.0, 132.9, 132.7, 128.9, 128.5, 128.4, 127.8, 122.6, 122.2, 94.1, 85.8, 66.5, 40.9.

**^11^B{^1^H} NMR** (128 MHz, DMSO*-d*_6_): δ [ppm] -1.15.

**^19^F{^1^H} NMR** (376 MHz, DMSO*-d*_6_): δ [ppm] 147.9.

**ESI-MS** calc. for C_116_H_80_N_8_O_8_Pd_2_B_3_F_12_ [M-BF_4_]^+^: *m/z* = 2187.4331; found: *m/z* = 2187.4243.

**IR** (ATR): [cm^-1^] 417, 519, 544, 669, 694, 745, 816, 951, 1049, 1153, 1196, 1319, 1420, 1456, 1487, 1506, 1541, 1558, 1732, 2222, 2363.


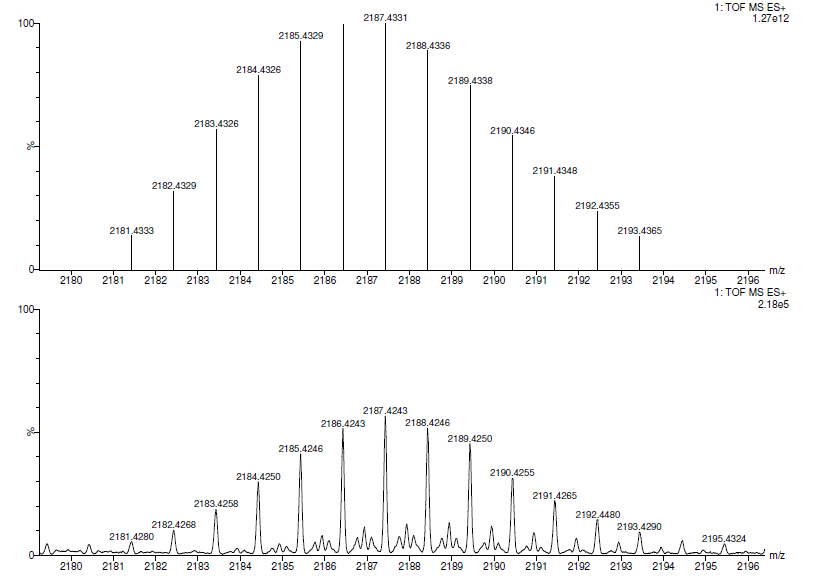


**Figure S3.** Comparison between the theoretical isotopic pattern (top) and the experimental pattern (bottom) of [**BnC1.BF_4_**-BF_4_]^+^.

**Cage C2**: 3-(3,5-bis(3-ethynylpyridine)phenyl)-propionic acid (**L2**) (35 mg, 0.1 mmol, 4 eq.) and palladium(II) nitrate dihydrate (13mg, 0.05 mmol, 2 eq.) were added to DMSO (5 mL) to form cage **C2** (30 mg, 16 µM, 65%).

**^1^H NMR** (400 MHz, DMSO*-d*_6_): δ [ppm] 12.17 (bs, 4H, H_COOH_), 9.72 (s, 8H, H_a_), 9.38 (d, J = 5.6 Hz, 8H, H_b_), 8.24 (d, J = 8.0, 8H, H_d_), 7.82 (t, J = 7.5 Hz, 8H, H_c_), 7.77 (s, 4H, H_e_), 7.60 (s, 8H, H_f_), 2.85 (t, J = 6.9 Hz, 8H, H_h_), 2.55 (b, 8H, H_g_).

**IR** (ATR): [cm^-1^] 411, 689, 810, 864, 949, 1026, 1107, 1192, 1225, 1319, 1418, 1506, 1584, 1717, 1734, 2220, 2361, 3076.

**
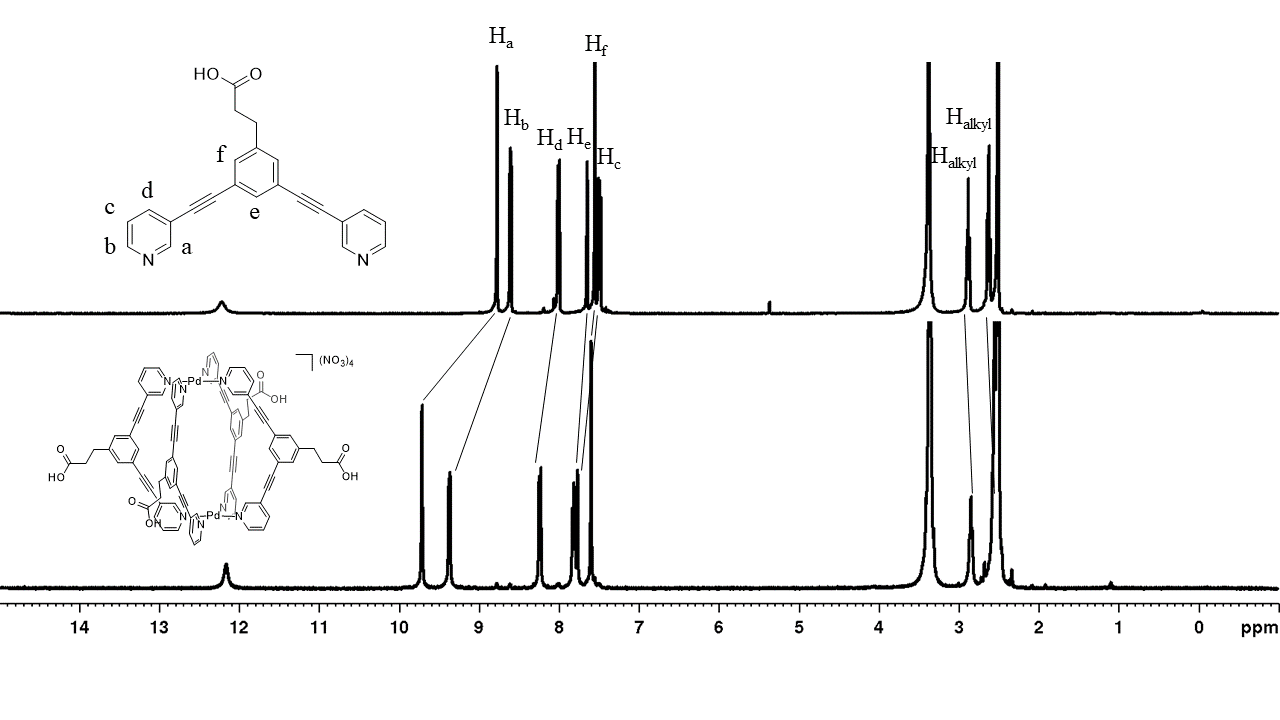
**

**Figure S4.** Example of monitoring metallacage formation by self-assembly. Stacked ^1^H NMR (DMSO*-d*_6_) spectra of **L2** (top) and **C2** (bottom) demonstrate the clear downfield shift of peaks H_a_ and H_b_ indicative of quantative cage formation.

**Cage C3**: 3,5-bis(3-ethynylpyridine)-1-(2-(2-methoxyethoxy)ethane)phenol **(L3)** (40 mg, 0.1 mmol, 4 eq.) and palladium(II) nitrate dihydrate (13 mg, 0.05 mmol, 2 eq.) were added to DMSO (3 mL) to form cage **C3** (30 mg, 16 µM, 83%).

**^1^H NMR** (400 MHz, DMSO*-d*_6_): δ [ppm] 9.71 (s, 9.70, 8H, H_a_), 9.37 (d, J = 5.0 Hz, 8H, H_b_), 8.26 (d, J = 8.2, 8H, H_d_), 7.81 (dd, J = 5.8, 7.9 Hz, 8H, H_c_), 7.51 (s, 2H, H_e_), 7.37 (s, 2H, H_e’_), 7.30 (s, 4H, H_f_), 7.07 (s, 4H, Hf’), 4.15 (t, J = 3.1 Hz, 8H, H_PEG_), 3.20 (s, 8H, H_PEG_), 2.89 (s, 8H, H_PEG_), 2.73 (s, 8H, H_PEG_).

**^13^C{^1^H} NMR** (101 MHz, DMSO*-d*_6_): δ [ppm] 159.2, 153.4, 151.0, 143.2, 127.8, 123.3, 122.7, 120.0, 94.2, 85.4, 71.7, 70.2, 69.2, 68.3, 58.5, 40.9.

**ESI-MS** calc for C_100_H_88_N_8_O_12_Pd_2_N_2_O_6_ [M-2NO_3_]^3+^: *m/z* = 644.4875; found: *m/z* = 644.4838. Calc for C_100_H_84_N_8_O_12_Pd_2_ [M-4NO_3_-4H]^4+^: *m/z* = 451.6159; found: *m/z* =451.6234.

**IR** (ATR): [cm^-1^] 694, 814, 864, 947, 1026, 1061, 1105, 1194, 1227, 1258, 1420, 1508, 1541, 1578, 1684, 1717, 2222, 2332, 2359, 2824, 2878, 2922.


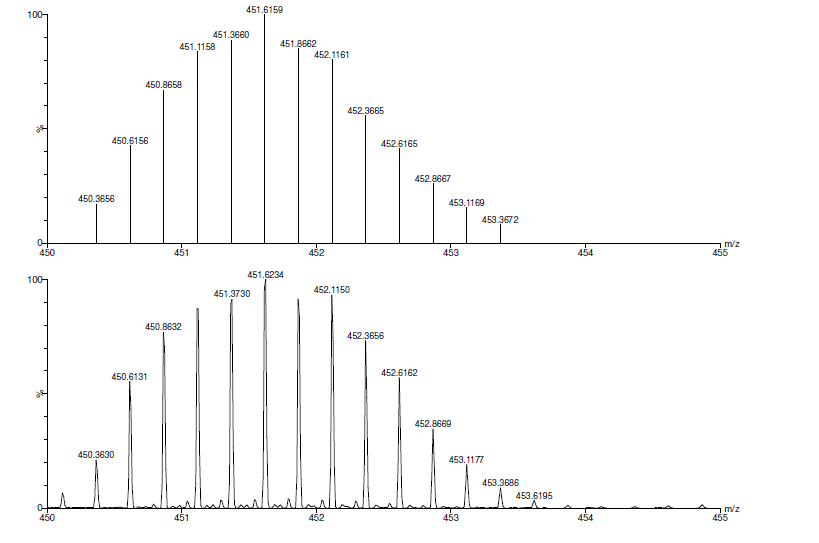


**Figure S5.** Comparison between the theoretical isotopic pattern (top) and the experimental isotopic pattern (bottom) of [**C3**-4NO_3_-4H]^4+^.

**Cage C4**: 3,5-bis(3-ethynylpyridine)-4-benzyl(thio-β-D-glucose tetraacetate) **(L4)** (66 mg, 0.1 mmol, 4 eq.) and palladium(II) nitrate dihydrate (13 mg, 0.05 mmol, 2 eq.) were added to DMSO (5 mL) to form cage **C4** (68 mg, 22 µM, 88%).

**^1^H NMR** (400 MHz, DMSO*-d*_6_): δ [ppm] 9.74 (d, J = 1.4 Hz, 2H, H_a_), 9.39 (d, J = 5.7 Hz, 2H, H_b_), 8.22 (d, J = 8.2 Hz, 2H, H_d_), 7.83 (dd, J = 5.8, 7.8 Hz, 2H, H_c_), 7.77 (s, 1H, H_e_), 7.67 (s, 2H, H_f_), 5.04-4.74 (m, 4H), 4.16-3.80 (m, 5H), 1.96-1.86 (m, 12H, H_Ac_).

**^13^C{^1^H} NMR** (101 MHz, DMSO*-d*_6_): δ [ppm] 170.4, 169.9, 169.7, 169.6, 153.4, 151.1, 132.5, 132.0, 131.9, 129.3, 129.2, 122.7, 122.2, 94.3, 85.5, 81.7, 74.7, 73.5, 70.0, 68.8, 62.6, 20.9, 20.8, 20.7.

**ESI-MS** Calc. for C_140_H_128_N_8_O_36_S_4_Pd_2_N_2_O_6_ [M-2NO_3_]^3+^: *m/z* = 988.8479; found: *m/z* = 988.8367.

**IR** (ATR): [cm^-1^] 694, 814, 864, 937, 1024, 1061, 1105, 1194, 1227, 1258, 1420, 1456, 1506, 1541, 1558, 1578, 1636, 1684, 1717, 2222, 2328, 2342, 2359, 2822, 2874, 2924, 3734.

**
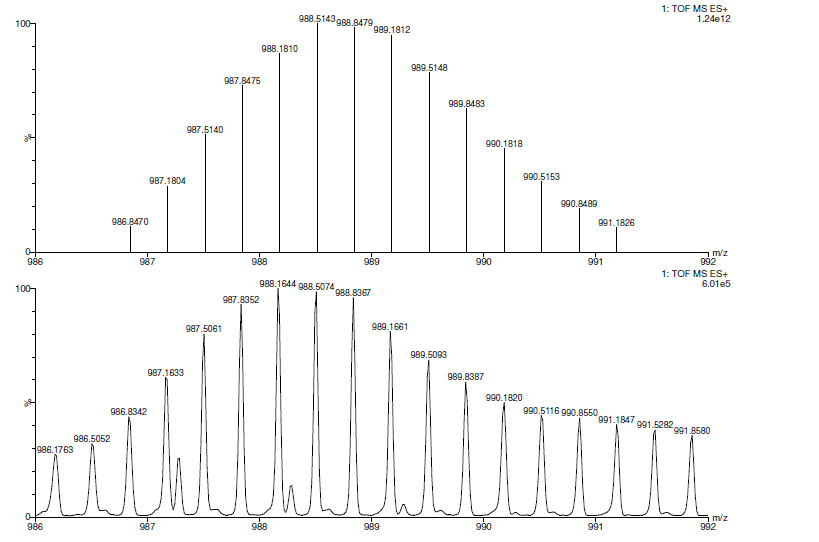
**

**Figure S6.** Comparison between the theoretical isotopic pattern (top) and the experimental isotopic pattern (bottom) of [**C4**-2NO_3_]^3+^.

**Cage C5:** 3,5-bis(3-ethynylpyridine)-4-benzyl(thio-β-D-glucose) **(L5)** (49 mg, 0.1 mmol, 4 eq.) and palladium(II) nitrate dihydrate (13 mg, 0.05 mmol, 2 eq.) were added to DMSO (5 mL) to form cage **C5** (41 mg, 17 µM, 68%).

**^1^H NMR** (400 MHz, DMSO*-d*_6_): δ [ppm] 9.75 (s, 2H, H_a_), 9.39 (d, J = 5.5 Hz, 2H, H_b_), 8.23 (d, J = 8.4, 2H, H_d_), 7.88-7.77 (m, 3H, H_c_, H_e_), 7.74 (s, 2H, H_f_), 4.19-3.51 (m, 9H).

**^13^C{^1^H} NMR** (101 MHz, DMSO*-d*_6_): δ [ppm] 153.3, 151.0, 143.2, 141.2, 134.5, 132.5, 132.0, 131.9, 129.3, 129.2, 127.8, 122.8, 122.1, 94.2, 85.5, 83.1, 81.7, 78.6, 77.2, 73.5, 70.6, 61.8, 61.7, 56.5.

**ESI MS** calc. for C_108_H_92_N_8_O_20_S_4_Pd_2_NO_3_ [M-3NO_3_-4H]^2+^: 1113.1729; found: *m/z* = 1113.1669. Calc. for C_108_H_92_N_8_O_20_S_4_Pd_2_ [M-4NO_3_-4H]^4+^: *m/z* = 541.5934; found: *m/z* = 541.5928.

**IR** (ATR): [cm^-1^] 413, 420, 457, 694, 814, 864, 945, 1024, 1061, 1105, 1194, 1227, 1258, 1314, 1339, 1420, 1456, 1506, 1541, 1558, 1578, 1636, 1684, 1717, 2222, 2342, 2359, 3628, 3649, 3821, 3838.


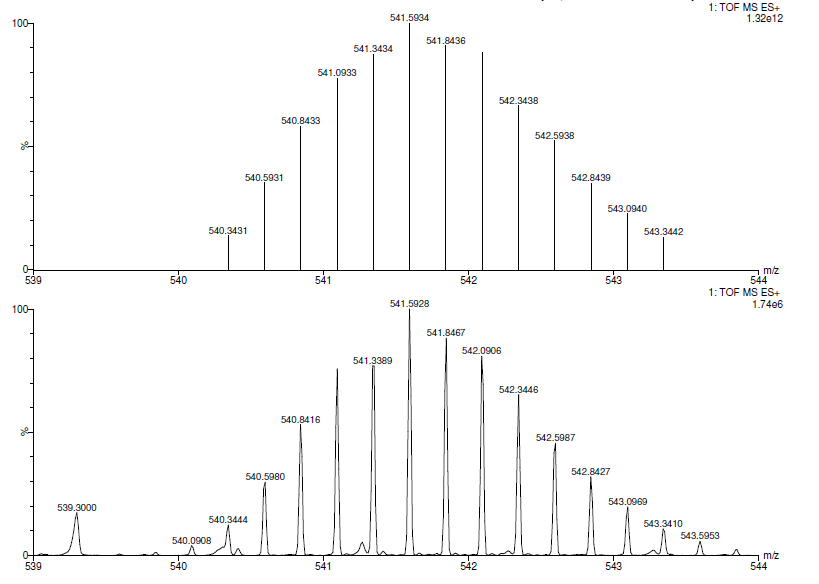


**Figure S7.** Comparison between the theoretical isotopic pattern (top) and the experimental isotopic pattern (bottom) of [**C5**-3NO_3_-4H]^4+^.

**Cage C6:** 2-(acetoxymethyl)-6-((1-(3,5-bis(pyridin-3-ylethynyl)benzyl)-1H-1,2,3-triazol-4-yl)methoxy)tetrahydro-2H-pyran-3,4,5-triyl triacetate **(L6)** (72 mg, 0.1 mmol, 4 eq.) and palladium(II) nitrate dihydrate (13 mg, 0.05 mmol, 2 eq.) were added to DMSO (5 mL) to form cage **C6** (72 mg, 22 µmol, 86%).

**^1^H NMR** (400 MHz, DMSO*-d*_6_): δ [ppm] 9.73 (s, 2H, H_a_), 9.40 (s, 2H, H_b_), 8.25 (d, J = 7.4 Hz, 2H, H_d_), 8.16 (s, 1H, H_h_) 7.90-7.78 (m, 2H, H_c_), 7.72 (s, 1H, H_e_), 7.67-7.61 (m, 2H, H_f_), 5.67 (s, 2H, H_g_), 5.32-4.39 (m, 5H, H_j_-H_n_), 4.25-3.81 (m, 2H, H_i_).

**ESI-MS** calc. for C_152_H_136_N_20_O_40_Pd_2_NO_3_ [M-3NO_3_-4H]^3+^: *m/z* = 1053.2489; found: *m/z* = 1053.1826.

**IR** (ATR): [cm^-1^] 412, 419, 473, 494, 617, 664, 718, 853, 1043, 1092, 1171, 1215, 1238, 1277, 1387, 1414, 1435, 1506, 1541, 1657, 1755.


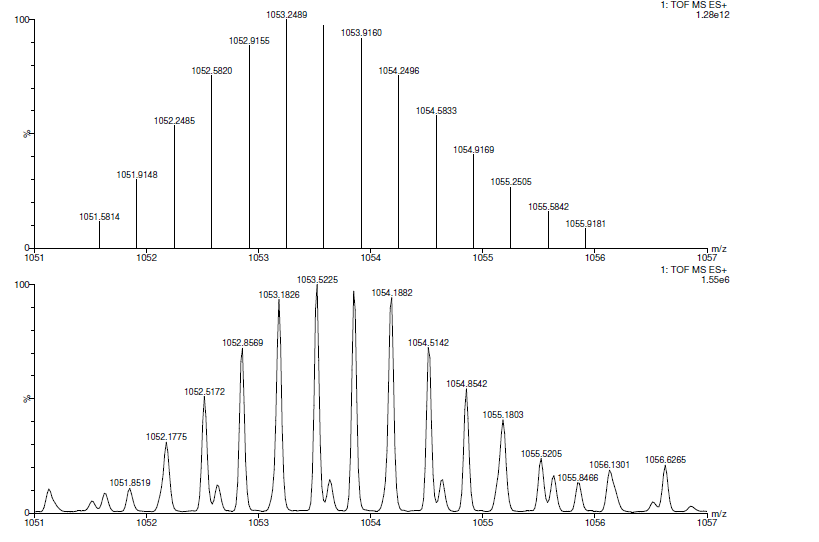


**Figure S8.** Comparison between the theoretical isotopic pattern (top) and the experimental isotopic pattern (bottom) of [**C6**-3NO_3_-4H]^3+^.

**Cage C7:** 2-((1-(3,5-bis(pyridin-3-ylethynyl)benzyl)-1H-1,2,3-triazol-4-yl)methoxy)-6-(hydroxymethyl)tetrahydro-2H-pyran-3,4,5-triol **(L7)** (55 mg, 0.1 mmol, 4 eq.) and palladium(II) nitrate dihydrate (13 mg, 0.05 mmol, 2 eq.) were added to DMSO (5 mL) to form cage **C7** (38 mg, 14 µmol, 57%).

**^1^H NMR** (500 MHz, DMSO*-d*_6_): δ [ppm] 9.69 (s, 8H, H_a_), 9.38 (s, 8H, H_b_), 8.26 (s, 8H, H_d_), 8.21 (s, 4H, H_h_), 7.96 (s, 4H, H_e_), 7.56 (s, 8H, H_f_), 7.50 (dd, J = 4.8, 7.4 Hz, 8H, H_c_), 5.73 (s, 8H, H_g_), 5.76 (s, 8H, H_g_), 5.05 (d, J = 5.4 Hz, 4H, H_OH_), 4.97-4.91 (m, 12H, H_OH_), 4.83 (d, J = 11.9 Hz, 4H, H_i_), 4.65 (d, J = 11.9 Hz, 4H, H_i’_), 3.74-3.63 (m, 8H, H_j_) 3.22-2.90 (m, 20H, H_Glucose_).

**ESI MS** calc. for C_120_H_108_N_20_O_30_Pd_2_NO_3_ [M-3NO_3_]^2+^: *m/z* = 1293.2848; found: *m/z* = 1293.1163.

**IR** (ATR): [cm^-1^] 411, 687, 754, 808, 1020, 1070,1190, 1271, 1319, 1506, 1541, 1558, 1653, 1717, 2324, 2359, 3649, 3854.

**
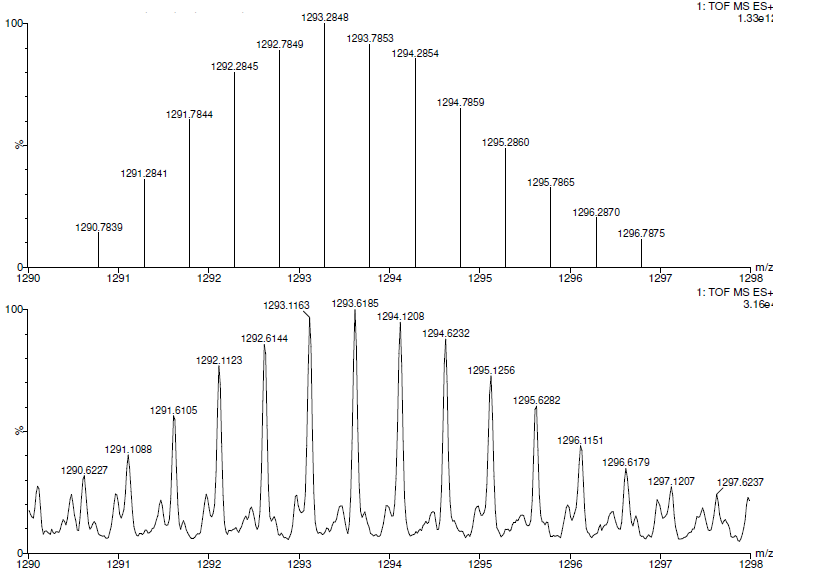
**

**Figure S9.** Comparison between the theoretical isotopic pattern (top) and the experimental isotopic pattern (bottom) of [**C7**-3NO_3_]^2+^.

**II. Encapsulation studies**

**General method**

**^1^H NMR Encapsulation studies**

The selected metallacage (4.4 µM, 1 eq.) was dissolved in 1 mL deuterated solvent (DMF*-d*_7_, MeCN*-d*_3_) and a ^1^H NMR spectrum was recorded. Cisplatin (8.8 µM, 2 eq.) was added to the NMR tube and the deuterated solution was stirred for 10 min. The ^1^H NMR spectrum was recorded and the spectra compared to evaluate any chemical shifts.

**^195^Pt NMR Encapsulation studies**

Cisplatin (2 mg, 7 µM, 1 eq.) was dissolved in DMF (0.5 mL). This solution was added to an NMR tube with a capillary tube insert of DMF*-d*_7_ and the^195^Pt NMR spectrum was recorded. The solution was removed from the NMR tube and **C1Bn.BF_4_** (16 mg, 7 µM, 1 eq.) was dissolved in the solution. The solution was transferred to an NMR tube with a capillary tube insert of DMF*-d*_7_ and the ^195^Pt NMR spectrum was recorded. The solution was removed from the NMR tube and cisplatin (2 mg, 7 µM, 1 eq.) was added. The solution was added to an NMR tube with a capillary tube insert of DMF*-d*_7_ and the ^195^Pt NMR spectrum was recorded.

**^1^H DOSY NMR Encapsulation study**

Cisplatin (1 mg, 3.5 µM, 1 eq) was dissolved in DMF-*d*_7_ (0.5 mL) and the ^1^H DOSY NMR spectrum was recorded at 25°C. To this solution metallacage **C1Bn.BF_4_** (8 mg, 3.5 µM, 1 eq.) was added and a ^1^H DOSY NMR spectrum was recorded at 25°C. To this solution cisplatin (1 mg, 3.5 µM, 1 eq.) was added and the ^1^H DOSY NMR spectrum was recorded at 25°C. The spectra were compared by calibration of the intensity of the DMF residual solvent signal (8.03 ppm (s, H_NCO_*_H_*); diffusion coefficient = - 8.75 x 10^10^ m^2^/s) to observe any quenching of the cisplatin signal.

**
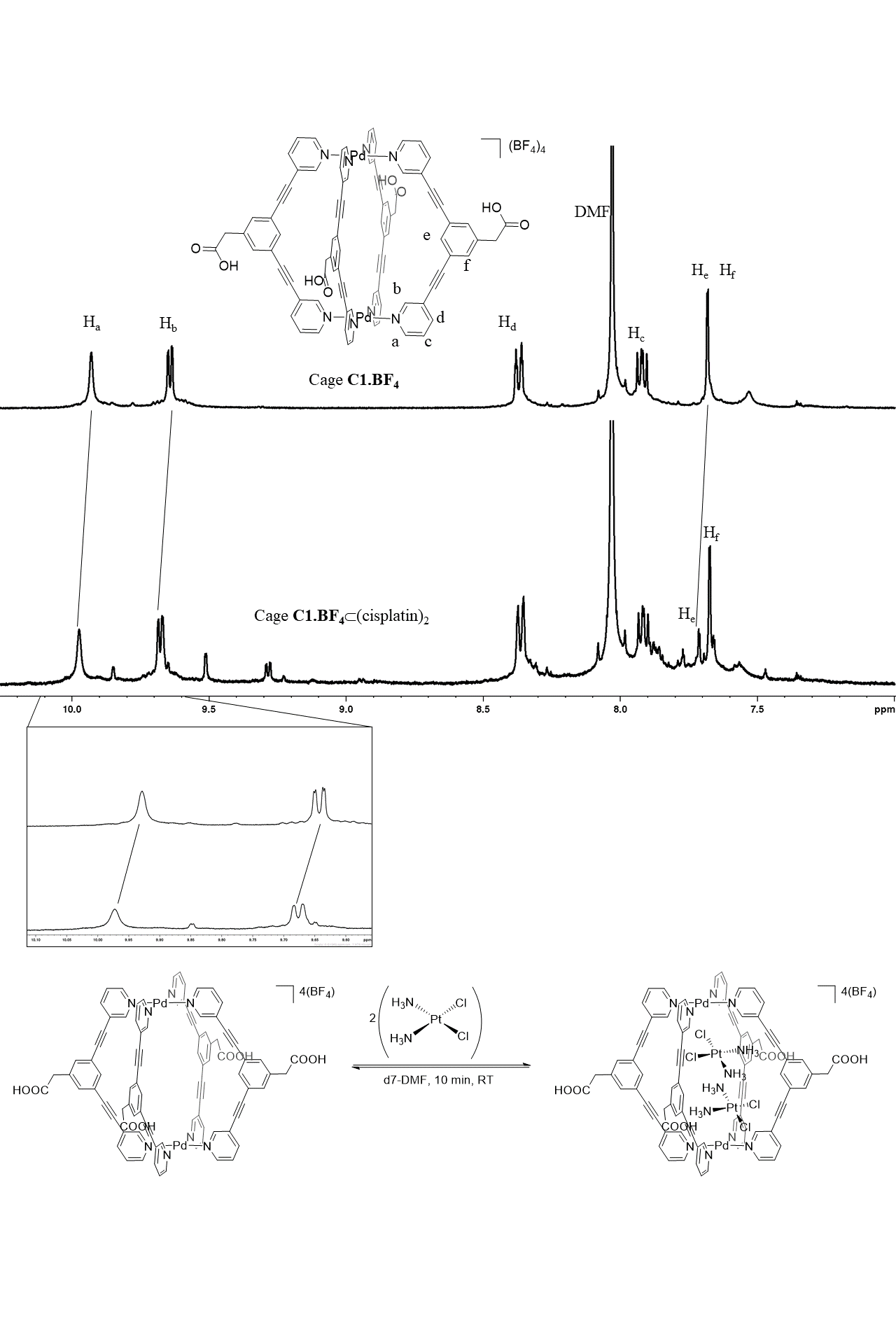
**

**Figure S10.** Stacked ^1^H NMR spectra in DMF*-d*_7_ of the aromatic region of: Top: Metallacage **C1.BF_4_**. Bottom: Metallacage **C1.BF_4_⊂(cisplatin)** after addition of 2 eq. cisplatin. Clear downfield shifts of proton peaks H_a_ (δ = +0.043 ppm) and H_b_ (δ = +0.033 ppm), consistent with encapsulation of cisplatin.


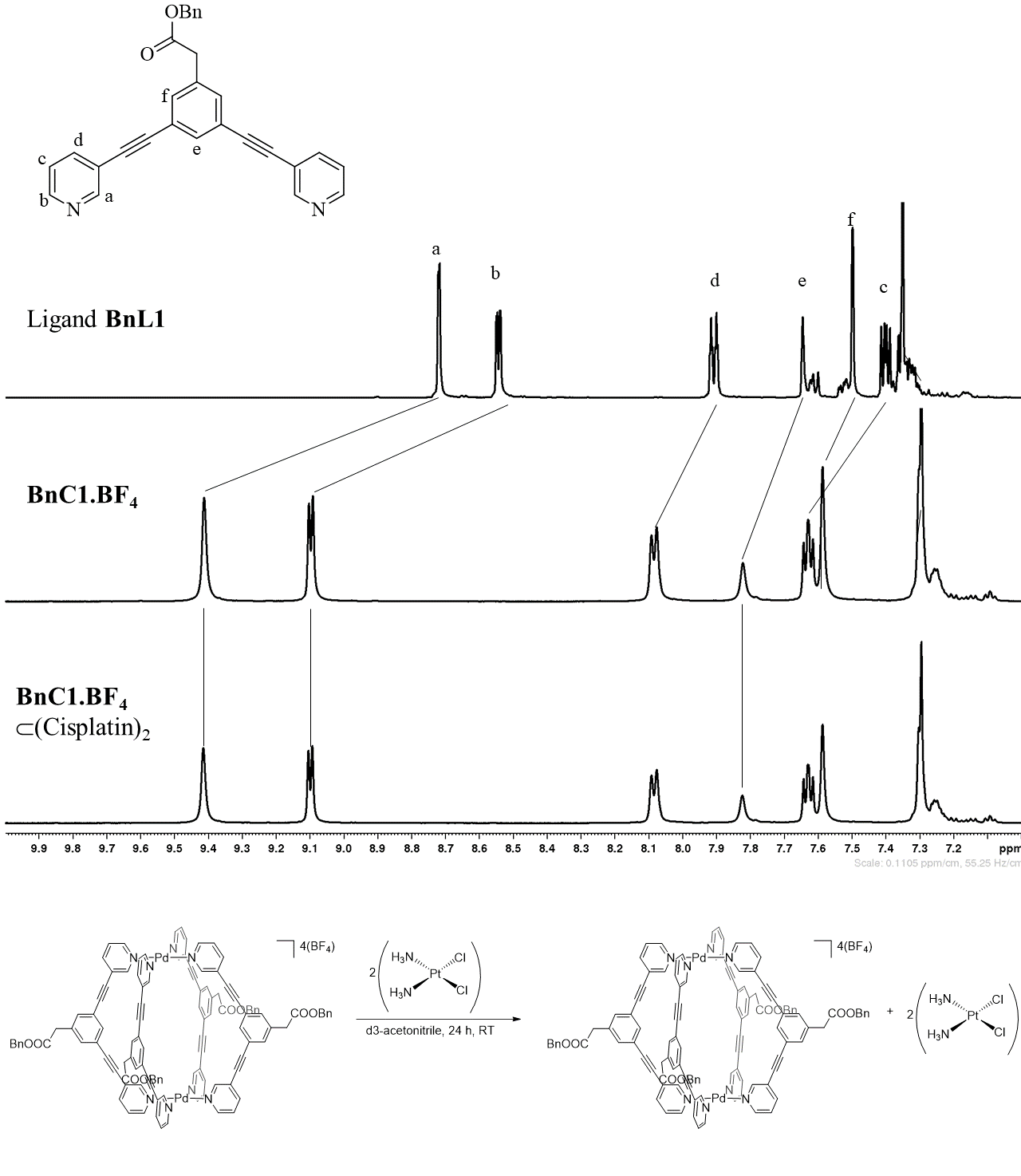


**Figure S11.** Stacked ^1^H NMR in MeCN*-d*_3_ of the aromatic region of: Top: Ligand **BnL1**; Middle: Metallacage **BnC1.BF_4_**; Bottom: Metallacage **BnC1.BF_4_ + 2 eq. cisplatin**. No chemical shifts are observed for any of the proton peaks, even after 24 h sonication at room temperature. This is consistent with no encapsulation of cisplatin.


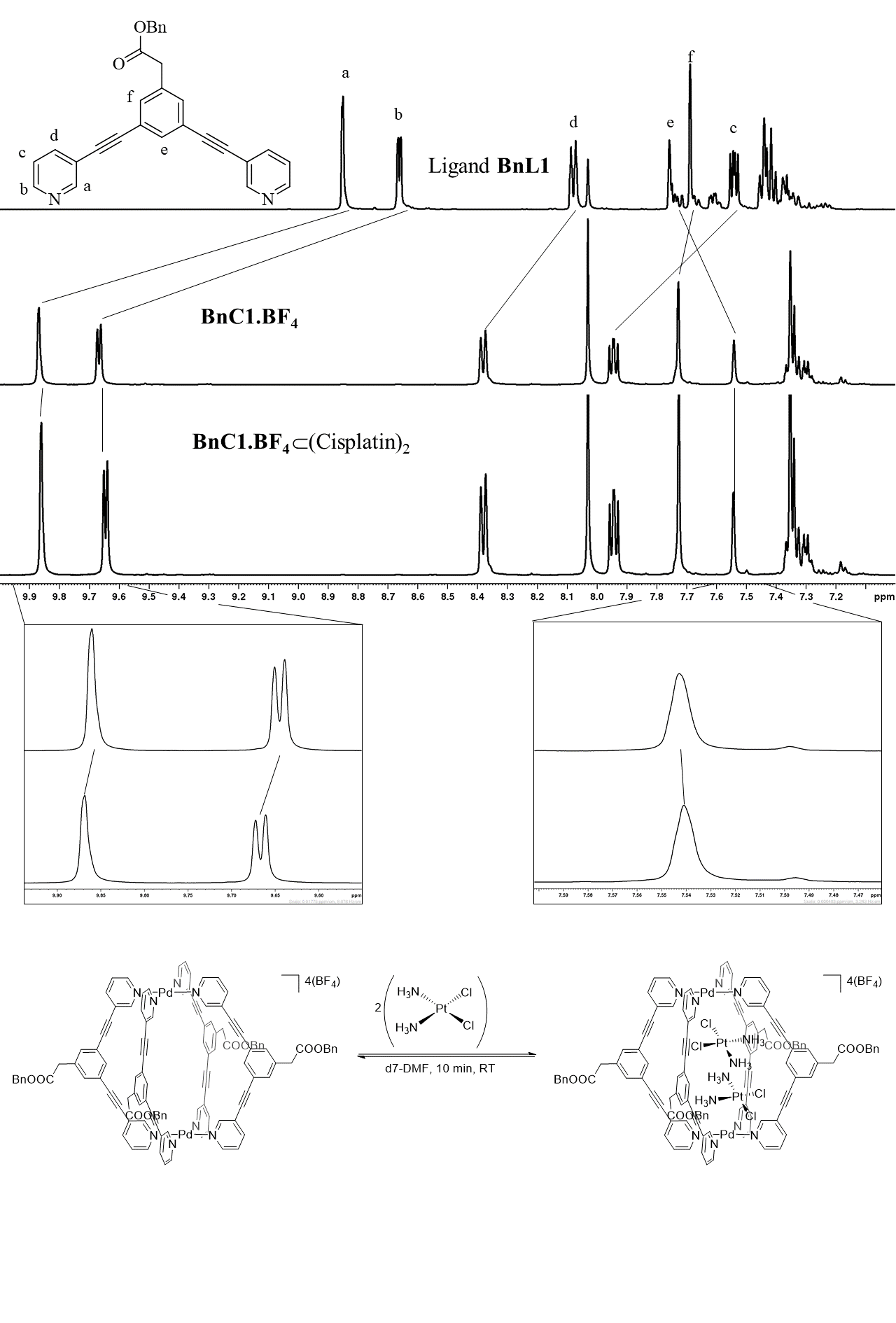


**Figure S12.** Stacked ^1^H NMR in DMF*-d*_7_ of the aromatic region of: Top: Ligand **L1Bn**; Middle: Metallacage **C1Bn.BF_4_**; Bottom: Metallacage **C1Bn.BF_4_⊂(cisplatin)_2_**. Downfield shifts are observed for cavity facing proton H_a_ (δ = +0.0079 ppm), as well as H_b_ (δ = +0.0226 ppm), and a small upfield shift for cavity facing proton He (δ = -0.0023 ppm). These chemical shifts are consistent with the encapsulation of cisplatin in metallacage **C1Bn.BF_4_**.


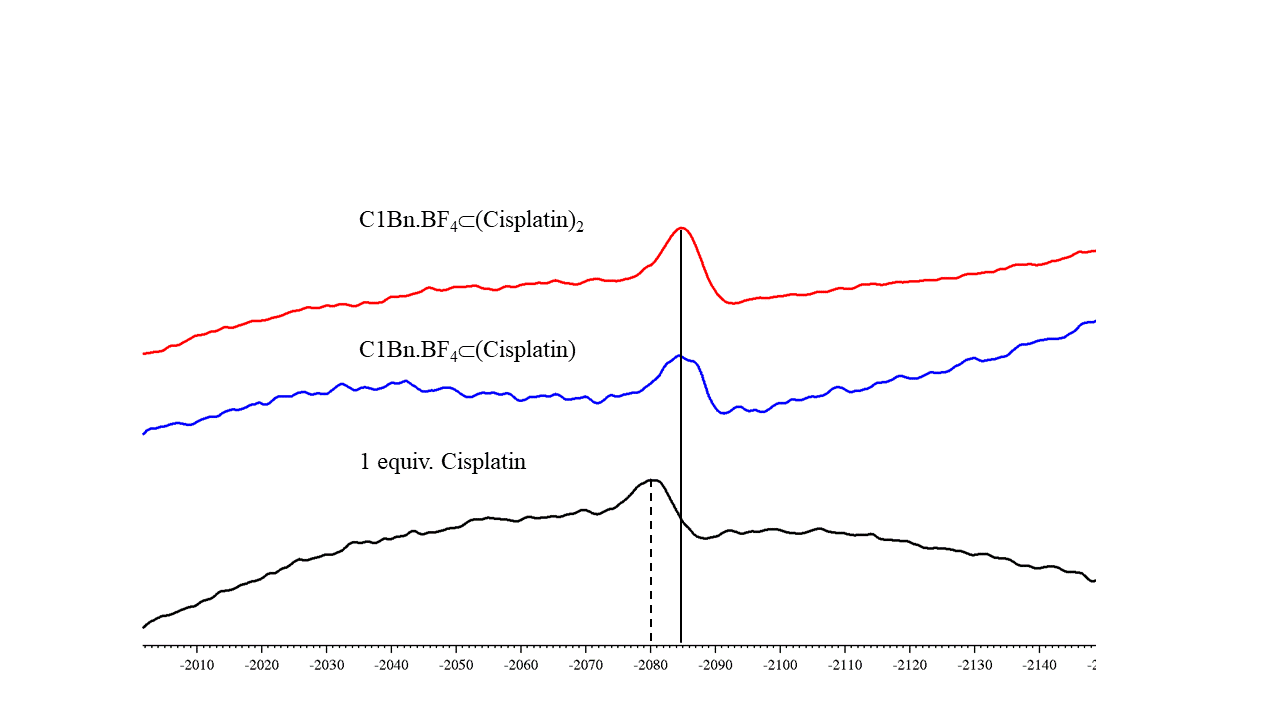


**
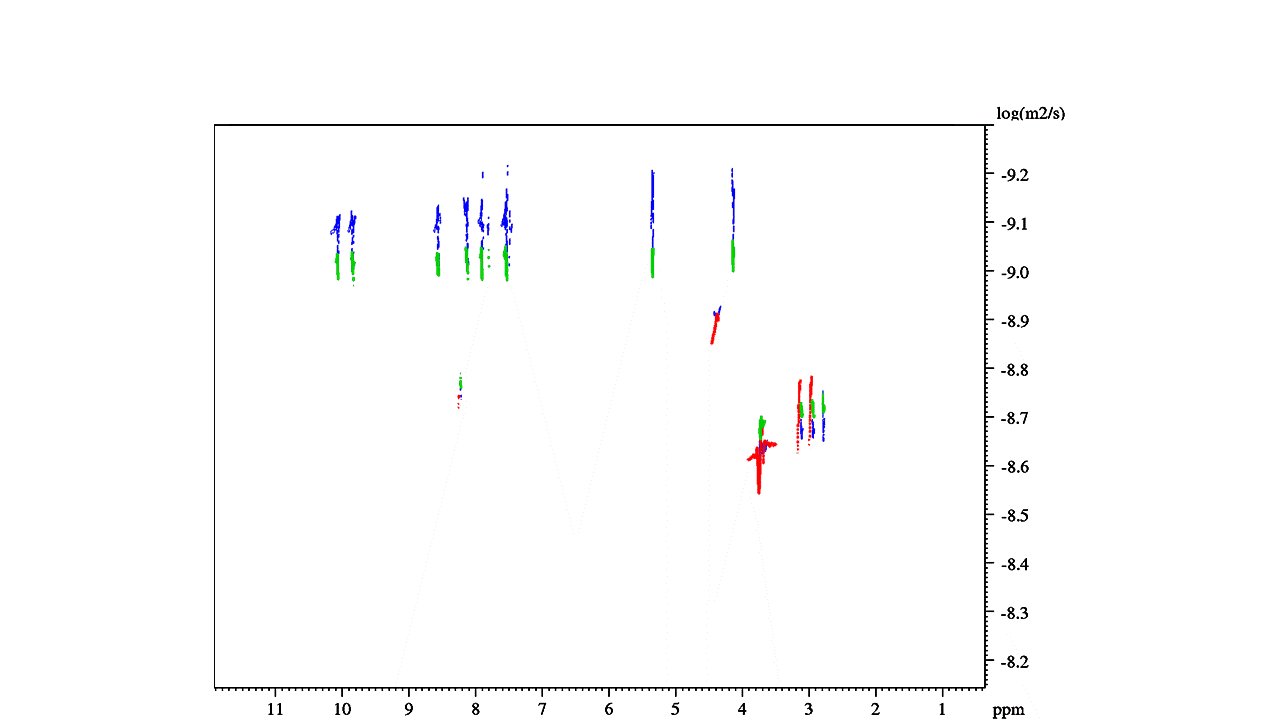
Figure S13.** Stacked ^195^Pt NMR spectra of DMF*-d*_7_ solutions of cisplatin alone or in the presence of metallacage **C1Bn.BF_4_** at different cisplatin:cage ratios. Bottom (black): 1 eq. of cisplatin (δ = -2080 ppm). Middle (blue): **C1Bn.BF_4_⊂(Cisplatin) 1:1**. Top (red): **C1Bn.BF_4_⊂(Cisplatin)_2_ 1:2** (δ = -2085 ppm). The upfield shifts of the platinum peak upon addition of the metallacage are consistent with encapsulation of cisplatin.

**Figure S14.** Stacked ^1^H DOSY NMR spectra showing: **Red**: ^1^H DOSY NMR spectrum of cisplatin (2 mg, 4.4 µM) in DMF-*d_7_* showing a strong signal for cisplatin (br, 4.18 ppm, H_NH3_; diffusion coefficient = -8.9 x 10^-10^ m^2^/s). **Green**: ^1^H DOSY NMR spectrum of cisplatin (2 mg, 4.4 µM) and **C1Bn.BF_4_** (8 mg, 4.4 µM, 1 eq.). The spectrum shows signals corresponding to metallacage **C1Bn.BF_4_** (9.87 ppm (s, 8H, H_a_), 9.65 (d, J = 3.5 Hz, 8H, H_b_), 8.37 (d, J = 7.4 Hz, 8H, H_d_), 7.93 (m, 8H, H_c_), 7.71 (s, 8H, H_f_), 7.61 (s, 4H, H_e_), 7.38-7.27 (m, 20H, H_phenyl_), 5.15 (s, 8H, H_alkyl_), 3.94 (s, 8H, H_alkyl_); diffusion coefficient = -9.1 x 10^-10^m^2^/s), but no signal for cisplatin (4.18 ppm, H_N_*_H_*_3_; diffusion coefficient = -8.9 x 10^-10^ m^2^/s). **Blue**: DOSY NMR spectrum of cisplatin (4 mg, 8.8 µM, 2 eq.) and metallacage **C1Bn.BF_4_** (8 mg, 4.4 µM, 1 eq.). The signal for cisplatin has reappeared, however, the intensity is less than that of 1 eq. of cisplatin in DMF. These results provide qualitative evidence of encapsulation of up to 2 eq. cisplatin.

**References**

**1**. Gottlieb, H. E., Kotlyer, V., Nudelman, A., (1997), “NMR chemical shifts of common laboratory solvents as trace impurities”, J. Org. Chem., 62:21, DOI: 10.1021/JO971176v

**2.** Rousseaux, S. A. L., Gng, J. Q., Haver, R., Odell, B., Claridge, T. D. W., Herz, L. M., *et al*., (2015) “Self assembly of Russian doll concentric porphyrin nanorings”, JACS, 137:39, DOI: 10.1021/jacs.5b07956

**3**. Gudipati, V., Curran, D. P., Wilcox, C. S., (2006) “Solution-​Phase Parallel Synthesis with Oligoethylene Glycol Sorting Tags. Preparation of All Four Stereoisomers of the Hydroxybutenolide Fragment of Murisolin and Related Acetogenins”, J. Org. Chem., 71:9, DOI:10.1021/jo060217x

**4**. Lewis, J. E. M., Elliott, A. B. S., McAdam, C. J., Gordon, K. C., Crowley, J. D., (2014) “ 'Click' to functionalise: synthesis, characterisation and enhancement of the physical properties of a series of exo- and endo-​functionalised Pd2L4 nanocages”, Chem. Sci., 5:5, DOI: 10.1039/c4sc00434e
